# An association between ATP7B expression and human cancer prognosis and immunotherapy: a pan-cancer perspective

**DOI:** 10.1186/s12920-023-01714-5

**Published:** 2023-11-30

**Authors:** Zhanzhan Zhang, Aobo Zhang, Yunpeng Shi, Zijun Zhao, Zongmao Zhao

**Affiliations:** 1 Department of Neurosurgery, The Second Hospital of Hebei Medical University, No. 215 Heping West Road, Xinhua District, Shijiazhuang, Hebei Province China; 2 Department of Neurosurgery, Sanbo Brain Hospital, Capital Medical University, No. 50, a pine in Xiangshan, Haidian District, Beijing, China; 3 Department of Neurosurgery, The Fourth Hospital of Hebei Medical University, No. 12 Jiankang Road, Chang’an District, Shijiazhuang, Hebei Province, China

**Keywords:** ATP7B, Pan-cancer, Immunotherapy, Cuproptosis, Prognosis

## Abstract

**Background:**

ATP7B is a copper-transporting protein that contributes to the chemo-resistance of human cancer cells. It remains unclear what the molecular mechanisms behind ATP7B are in cancer, as well as its role in human pan-cancer studies.

**Methods:**

Our study evaluated the differential expression of ATP7B in cancer and paracancerous tissues based on RNA sequencing data from the GTEx and TCGA. Kaplan–Meier and Cox proportional hazards regressions were used to estimate prognostic factors associated with ATP7B.The correlations between the expression of ATP7B and immune cell infiltration, tumor mutation burden, microsatellite instability and immune checkpoint molecules were analyzed. Co-expression networks and mutations in ATP7B were analyzed using the web tools. An analysis of ATP7B expression difference on drug sensitivity on tumor cells was performed using the CTRP, GDSC and CMap database.

**Results:**

ATP7B expression differed significantly between cancerous and paracancerous tissues. The abnormal expression of ATP7B was linked to prognosis in LGG and KIRC. Infiltration of immune cells, tumor mutation burden, microsatellite instability and immunomodulators had all been linked to certain types of cancer. Cancer cells exhibited a correlation between ATP7B expression and drug sensitivity.

**Conclusion:**

ATP7B might be an immunotherapeutic and prognostic biomarker based on its involvement in cancer occurrence and development.

**Supplementary Information:**

The online version contains supplementary material available at 10.1186/s12920-023-01714-5.

## Introduction

As of 2020, nearly 10.0 million cancer-related deaths would occur, and 19.3 million cancer cases will have been diagnosed [1]. As a result, cancer deaths and cancer burdens are increasing. Studies have also demonstrated the growth of tumors is dependent on the tumor microenvironment, which is complex, and the tumor-infiltrating immune cells are crucial to this environment [2, 3]. Cancer immunotherapy has gained a significant amount of traction in the treatment of cancer. Among them are checkpoint inhibitors, lymphocyte-activating cytokines, CAR T cells and other cellular therapies, protein-binding antibodies against co-stimulatory receptors, cancer vaccines, oncolytic viruses, and bispecific antibodies. In order to implement immunotherapies for cancer widely, the immune system must be controlled, as these therapies may cause autoimmunity and other serious adverse effects [4].

It has been demonstrated that metal levels can affect the protein expression involved in metal metabolism in a variety of cancers and tumor metastasis [5–7]. ‘Curotosis’ is the first type of copper-induced regulated cell death proposed by Tsvetkov et al., which presents the copper directly binds to lipoylated components of the tricarboxylic acid (TCA) cycle and leads to the aggregation of lipoylated protein and Fe-S cluster protein loss [8]. Then, proteotoxic stress occurs, leading to cell death [8]. The following 19 genes are involved in cuprotosis: NFE2L2, NLRP3, ATP7B, ATP7A, SLC31A1, FDX1, LIAS, LIPT1, LIPT2, DLD, DLAT, PDHA1, PDHB, MTF1, GLS, CDKN2A, DBT, GCSH, DLST [8]. Copper is delivered to the transporter ATP7A and ATP7B, whose function is the export of excess copper from cells [9, 10]. In contrast, SLC31A1 is responsible for intracellular copper uptake [11].

Copper metabolism may influence carcinogenic signaling pathways and anti-tumor medication resistance [7]. Overexpression of TMEM16A leaded to transcriptional activation of ATP7B expression, promoting vesicular isolation of platinum compounds and reducing their efficacy and sensitivity to cisplatin. This new explanation for the platinum resistance mechanism of tumors provided a new pathway for treating these tumors [12]. The ATP7B gene was involved in glioblastoma resistance to temozolomide [13]. ATP7B has been found to be highly expressed in human epidermoid carcinoma and prostate cancer patients treated with platinum drugs, and is associated with poor prognosis [14].

The tumor mutation burden (TMB) is a recognized immunotherapy biomarker that determines the prognosis of cancer patients treated with immune checkpoint inhibitors (ICIs). However, high TMB can better provide assistance for tumor immunotherapy and better serve the patient population [15, 16]. According to recent studies, copper death genes are associated with multiple cancer types and levels of immune cell infiltration and can serve as candidate genes for cancer diagnosis, prognosis, and treatment biomarkers [7, 17]. However, the molecular mechanism and regulation of ATP7B between pan-cancer and immunity remain a mystery, which may provide therapeutic targets for pan-cancer treatment and overcome chemotherapeutic drug resistance.

Using the Cancer Genome Atlas (TCGA), we examined how ATP7B expression correlated with prognosis and stage among different tumor types. Deep learning-based on ATP7B research offers a promising prospect in this study.

## Materials and methods

### Analyzing tumor databases for gene expression profiles and clinical information

Our study collected data on 33 solid tumors, including transcriptome profiling and clinical follow-up survival and staging (ACC, BLCA, BRCA, CESC, CHOL, COAD, ESCA, GBM, HNSC, KICH, KIRC, KIRP, LGG, LIHC, LUAD, LUSC, MESO, OV, PAAD, PRAD, READ, SARC, SKCM, STAD, TGCT, THCA, THYM, and UCEC) from the Cancer Genome Atlas (TCGA, https://portal.gdc.cancer.gov/repository) and the UCSC XENA (https://xena.ucsc.edu/) databases. As part of this study, Cancer Therapeutics Response Portal (CTRP) and Genomics of Drug Sensitivity in Cancer (GDSC) data from Gene Set Cancer Analysis (GSCA, http://bioinfo.life.hust.edu.cn/GSCA/#/drug) were combined to assess drug sensitivity. Through common gene expression signatures, we used CMap analysis to link genes, drugs, and disease status [18, 19]. We also used a human protein profile database to demonstrate the expression of copper poisoning in cancer tissues (Human Protein Atlas, HPA, http://www.proteinatlas.org/).

### The analysis of Cox regression, Kaplan–Meier analysis, and Nomogram construction

Multivariate cox proportional hazards regression and LASSO analysis were applyed to identify five cuprotosis-related feature genes, we developed a model for predicting glioma prognosis based on our study. To calculate risk scores, we calculated expression coefficients for each of the five genes: risk score = Exp(gene1) *Coef(gene1) + Exp(gene2) *Coef(gene2) + …Exp (gene n) *Coef (gene n). The median risk score was then used to divide patients into two groups. Subsequently, TCGA, Chinese Glioma Genome Atlas (CGGA), Gravendeel and Rembrandt cohorts were examined using Kaplan–Meier (K-M) curves and cox proportional hazards models. Identifying prognostic factors for glioma survival rates over 1, 2, and 3 years was the basis for developing the nomogram.

### ATP7B CpG methylation and prognosis

A new diagnostic model that uses DNA methylation to identify cancer occurrence and prognosis is emerging [20–22]. We used Methsurv (https://biit.cs.ut.ee/methsurv) to analyze the expression and prognostic relationship of a single CpG methylation site of ATP7B in 25 types of cancer [23].

### cBioPortal database and GeneMANIA analysis

Using the R package ‘ggpubr’, our study compared ATP7B expression in tumor cells with that in normal cells. The cBio Cancer Genomics Portal (cBioPortal) (http://www.cbioportal.org) was used to assess the ATP7B mutations (amplification, deep deletion, and missense mutations) for patients with various types of cancer. Our gene–gene interaction networks were created using GeneMANIA (http://www.genemania.org) [24].

### Survival and prognosis analysis of ATP7B in pan-cancer

In single-sample GSEA studies (ssGSEA), enrichment scores were calculated separately for each pair of samples and gene sets. The default parameters of the GSVA, limma and GSEABase R package were used to implement ssGSEA. By using the boxplot R package, we were able to illustrate the variation in the activity of ATP7B expression across cancer types. A Wilcoxon rank sum test was used to determine whether two groups displayed differential expression.

We used the ‘survival’ and ‘forest’ packages to calculate hazard ratios and cox *p* values for disease-specific survival (DSS), overall survival (OS), disease-free interval (DFI) and progression-free interval (PFI) in TCGA pan-cancer analyses. With R4.1.3, we used the packages ‘survival’, ‘survminer’ to classify tumor types based on median expression levels of ATP7B, and drew the K-M survival curves.

### Different types of tumors harbour different immune characteristics correlated with ATP7B expression

Immune infiltration within the immune microenvironment affected cancer prognosis, treatment, and progression. Immune infiltration was determined immune score and stromal score using the ESTIMATE algorithm, followed by a correlation analysis of immune infiltration with ATP7B expression.

We analyzed molecular interactions between tumors and the immune system with the Tumor Immune Estimation Resource (TIMER, https://cistrome.shinyapps.io/timer/) [25]. An analysis of the correlation between ATP7B and 22 immune cell types was carried out using CIBERSORT. ATP7B expression and immune infiltration were compared using spearman correlation analysis.

### An analysis of ATP7B gene enrichment in pan-cancer cells

To enrich functional and pathway information, gene set enrichment analysis (GSEA) was performed to explore the functional significance of ATP7B expression in tumors. Data sets for gene ontology (GO) and Kyoto Encyclopedia of Genes and Genomes (KEGG) biological processes were retrieved from the GSEA website (Molecular Signatures Database, MSigDB, http://www.broadinstitute.org/gsea/) [26–28]. As part of this study, we used limma, org.Hs.eg.db, clusterProfiler, and enrichplot to conduct GSEA.

### A pan-cancer study using tumor mutation burden and microsatellite instability

This study used spearman correlation coefficient to investigate the relationship between the expression of ATP7B and tumor mutation burden (TMB) or microsatellite instability (MSI). The radar chart was created using the fmsb package.

### Drug susceptibility analysis

Incorporation of GDSC and CTRP data was undertaken in this study to determine drug sensitivity and gene expression profiles as part of the common antitumor drug sensitivity analysis. Using pearson correlation analysis, we determined the relationship between drug IC50 and gene expression. As a second step, we searched the CMap website to select small molecule drugs associated with genes and diseases, and then compared them with the previous GDSC and CTRP results to find common drugs [18, 19].

### In vitro culture of cells

Human astrocytes (HAs) were cultured with HA culture medium (Astrocyte Medium) (both from ScienCell Research Laboratories, Inc. (San Diego, CA, USA)). And the glioma cell lines (SHG44, U251 and T98G) were obtained from Procell Life Science & Technology Co., Ltd. (Wuhan, China). Roswell Park Memorial Institute (RPMI) -1640 medium (Gibco, Thermo Fisher Scientific, Shanghai, China) was used as the basal culture medium of U251 and SHG44 cells, while the basal culture medium of T98G was minimal essential medium (MEM) (Gibco, Thermo Fisher Scientific, Shanghai, China). SHG44 and U251 cells were cultured with RPMI-1640 medium supplemented with 10% fetal bovine serum (FBS) and 1% penicillin–streptomycin (P/S) (Biological Industries at Sartorius, Kibbutz Beit-Haemek, Israel). T98G cells were maintained in the presence of MEM, 10% FBS and 1% P/S. HAs, SHG44, U251, and T98G cells were cultured in a sterile cell incubator at 37 °C with 5% CO_2_.

### RT-PCR

Total RNA from HA, U251, and T98G cells were extracted using Superbrilliant™ 6 min High-quality RNA Extraction Kit (Zhongshi Gene Technology, Tianjin, China, Cat. No.: ZSM11005). cDNA synthesis was carried out using the Supersmart ™ 6 min 1st Strand cDNA Synthesizer Kit (Zhongshi Gene Technology, Cat. No.: ZS-M14003). QPCR was performed with Supersmart 5xFast SYBR Green qPCR Mix Kit (Zhongshi Gene Technology, Cat. No.: ZS-M13001) on Bio-Rad Laboratories CFX Connect (TM) Real-time PCR Detection System (Bio-Rad, Hercules, CA, USA). The primers were obtained from Thermo Scientifific (Shanghai, China), and included those amplifying ATP7B (forward 5′-AGGTGGATGCAGCCTCCGTTTA-3′, reverse 5′-AT.

GAGCCGTTCACAGTTTCCCG-3′); Relative mRNA levels were calculated using the 2_-△△Ct_ method. Each experiment was carried out independently three times.

### Western Blot

Low-grade glioma tissue and high-grade glioma tissue were collected. Cleavage ina radioimmunoprecipitation (RIPA) buffer containing protease and phosphatase inhibitors (Solarbio Science and Technology, Beijing, China). The lysate was subjected to 10% sodium dodecyl sulfate polyacrylamide gel electrophoresis (SDS-PAGE, Solarbio Science and Technology). And then transferred to polyvinylidene fluoride (Millipore, Billerica, Massachusetts, USA). The membrane was blocked with 5% skim milk for 2 h and incubated overnight at 4 °C with primary antibodies against the following proteins: ATP7b (Abways Biotechnology, Shanghai, China, catalog number: CY8616), as well as GAPDH (Abways Biotechnology, Shanghai, China, catalog number: P04406). Subsequently, a secondary goat anti-rabbit IgG antibody (Abways Biotechnology, catalog number: AB0101) was added to the membrane and incubated at 25 °C for 1 h. Finally, immunoreactive proteins were detected using ECL Western blot substrates (Solarbio Science and Technology, catalog number: PE0010) and analyzed using the BioRad ChemiDoc imaging system (Bio-Rad, USA).

### Statistical analysis

The Kruskal–Wallis test was employed to compare normal samples and tumor subtypes of samples. Differences in ATP7B expression levels between cancerous and para-cancerous tissues were assessed through the use of Wilcoxon rank-sum tests. More than two groups were compared using analysis of variance [29–31]. Survival analysis was conducted using cox-proportional hazard regression, while Log-rank tests were utilized to compare survival times derived from Kaplan–Meier survival curves. Statistical analysis was performed using R software (version 4.1.3), and statistical significance was determined by a *P* value < 0.05.

## Result

### Using tumor databases to obtain gene expression profiles and clinical data

**Fig. 1:**
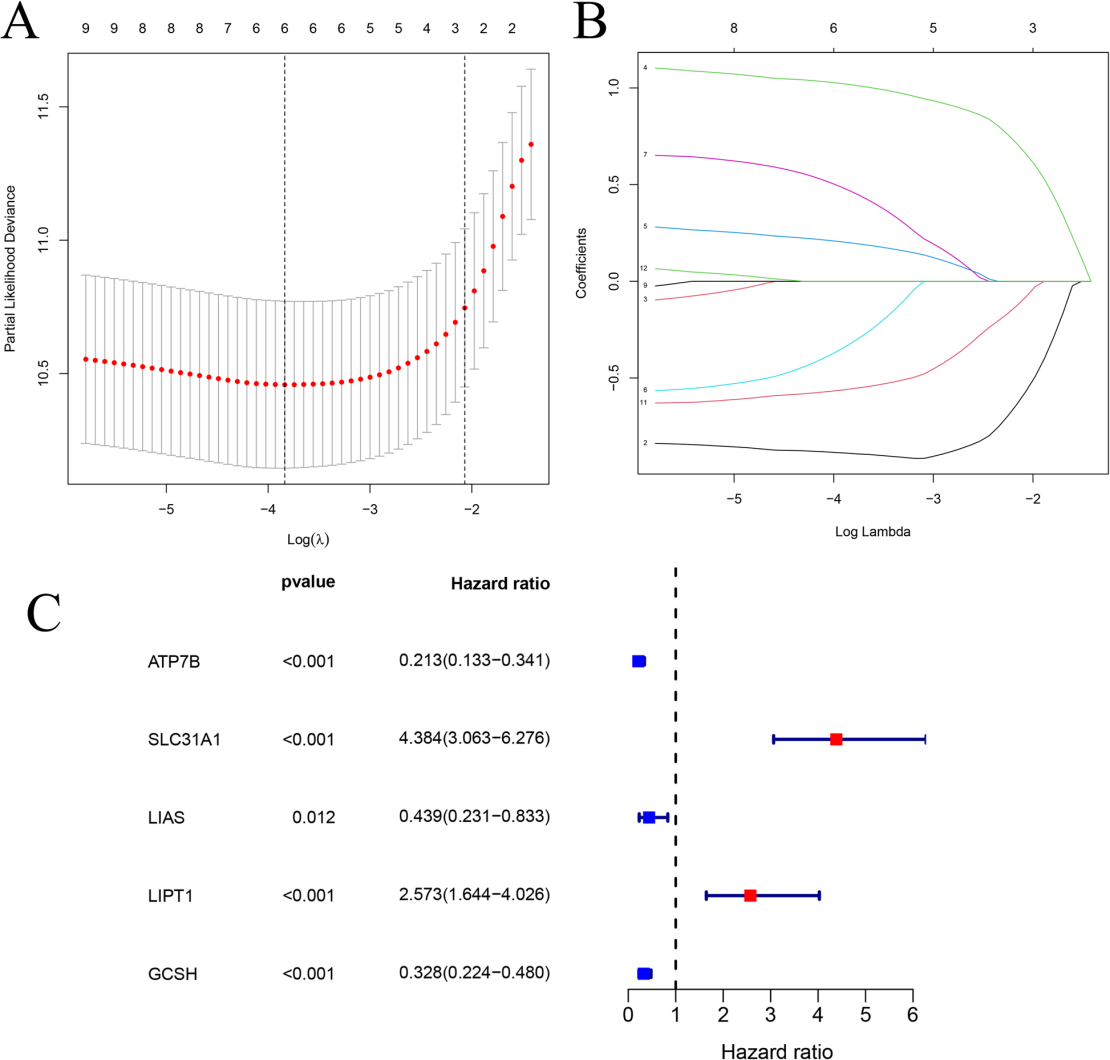
Prognostic risk model for cuproptosis-related genes. **A** LASSO coefficient profiles of 5 differentially expressed genes associated with cuproptosis. **B** The LASSO model used 10-fold cross-validation with minimum criteria to select the parameter (&lambda). **C** A forest plot showed the five genes associated with cuproptosis

**Fig. 2:**
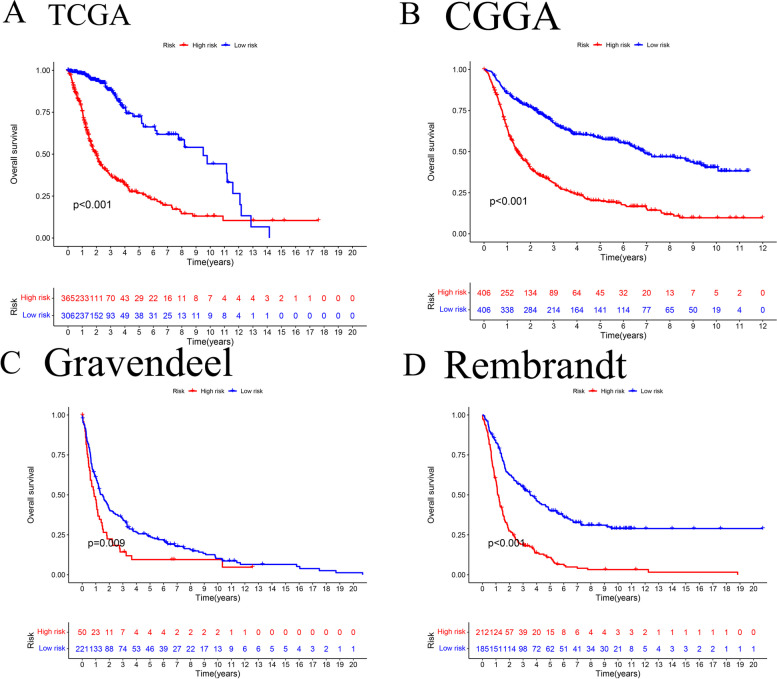
Kaplan–Meier survival curve for glioma patients expressing cuproptosis-related genes. groups at high risk had lower overall survival than those at low risk in the TCGA (**A**), CGGA (**B**), Gravendeel (**C**) and Rembrandt studies (**D**). (Kaplan–Meier survival curves depict blue as low risk and red as high risk.)

**Fig. 3:**
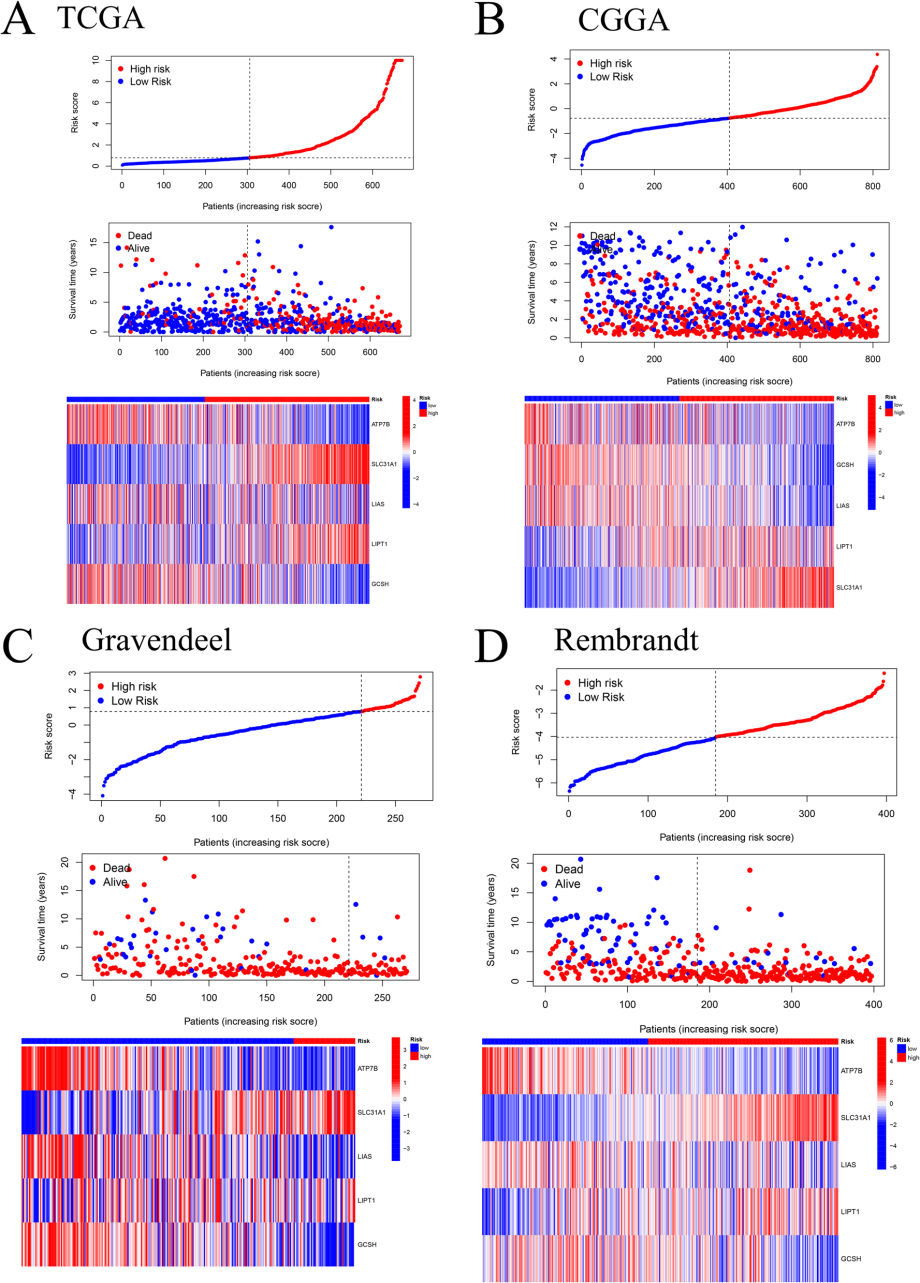
The risk model in different groups and its prognosis. Gene-risk scores, survival status, and heatmaps of gene expression were depicted in a model of prognosis risk for five cuproptosis-related genes across three studies in the TCGA (**A**), CGGA (**B**), Gravendeel (**C**), and Rembrandt (**D**) cohorts. (Survival state diagrams depict blue as alive and red as dead. Gene risk score diagrams and heatmaps depict blue as low risk and red as high risk.)

In this study, we developed a prognostic model to estimate the survival risk score by considering the expression levels and relative contributions of the five genes associated with cuproptosis, including ATP7B, SLC31A1, LIAS, LIPT1, GCSH (Fig. 1A-C). Furthermore, we evaluated the significance of risk scores for overall survival (OS) through K-M survival curve analyses (*p* < 0.05). The OS of the low-risk group was higher than those of the high-risk group in the TCGA, CGGA, Gravendeel, and Rembrandt cohorts (Fig. 2A-D). Risk heatmaps, risk curves, and scatter plots provided information on how glioma risk scores related to survival status, revealing that mortality rates rised with higher risk scores (Fig. 3 A-D).

### An analysis of the prognostic effect of ATP7B in gliomas

**Fig. 4:**
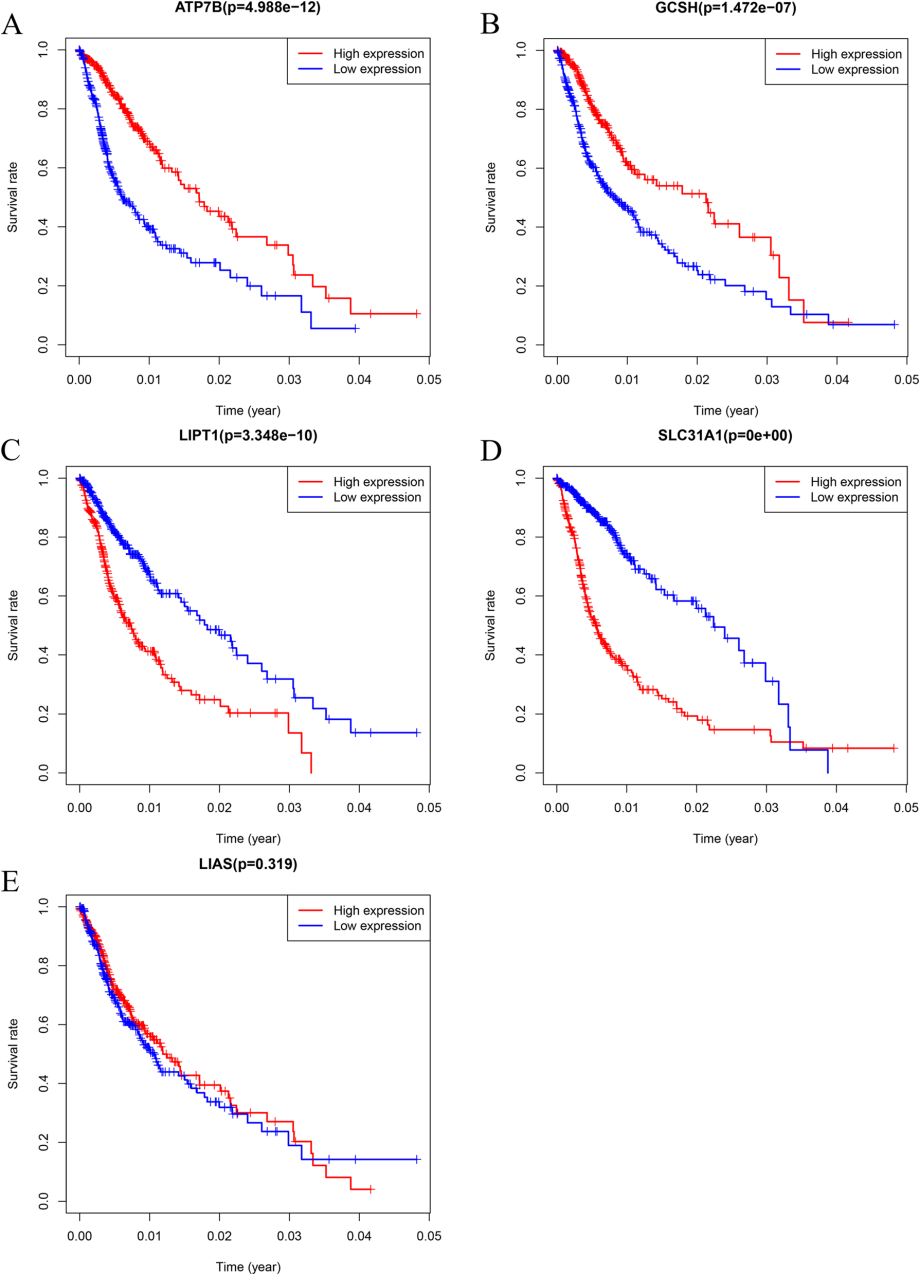
The Kaplan–Meier survival curves of patients with low and high expression populations of 5 genes related to cuprotosis were analyzed. **A**, **B** Kaplan–Meier survival curves indicated that patients with high expression of ATP7B and GCSH had significantly higher survival rate than patients with low expression. **C**, **D** Kaplan Meier survival curves indicated that patients with low expression of LIPT1 and SLC31A1 had significantly higher survival rate than patients with high expression. E. LIAS expression was not associated with prognosis in patients with glioma. (Kaplan–Meier survival curves depict blue as low expression and red as high expression.)

The K-M survival curve of glioma patients was plotted using the survival R software package to evaluate the prognostic value of 5 cuproptosis-related characteristic genes overexpression(*p* < 0.05) (Fig. 4). Survival rates were higher among patients who had overexpression of ATP7B and GCSH (Fig. 4A, B). The overexpression of LIPT1 and SLC31A1 predicted poor prognosis in patients (Fig. 4C, D). Patients with glioma did not show any prognostic value for LIAS expression (Fig. 4E).

**Fig. 5:**
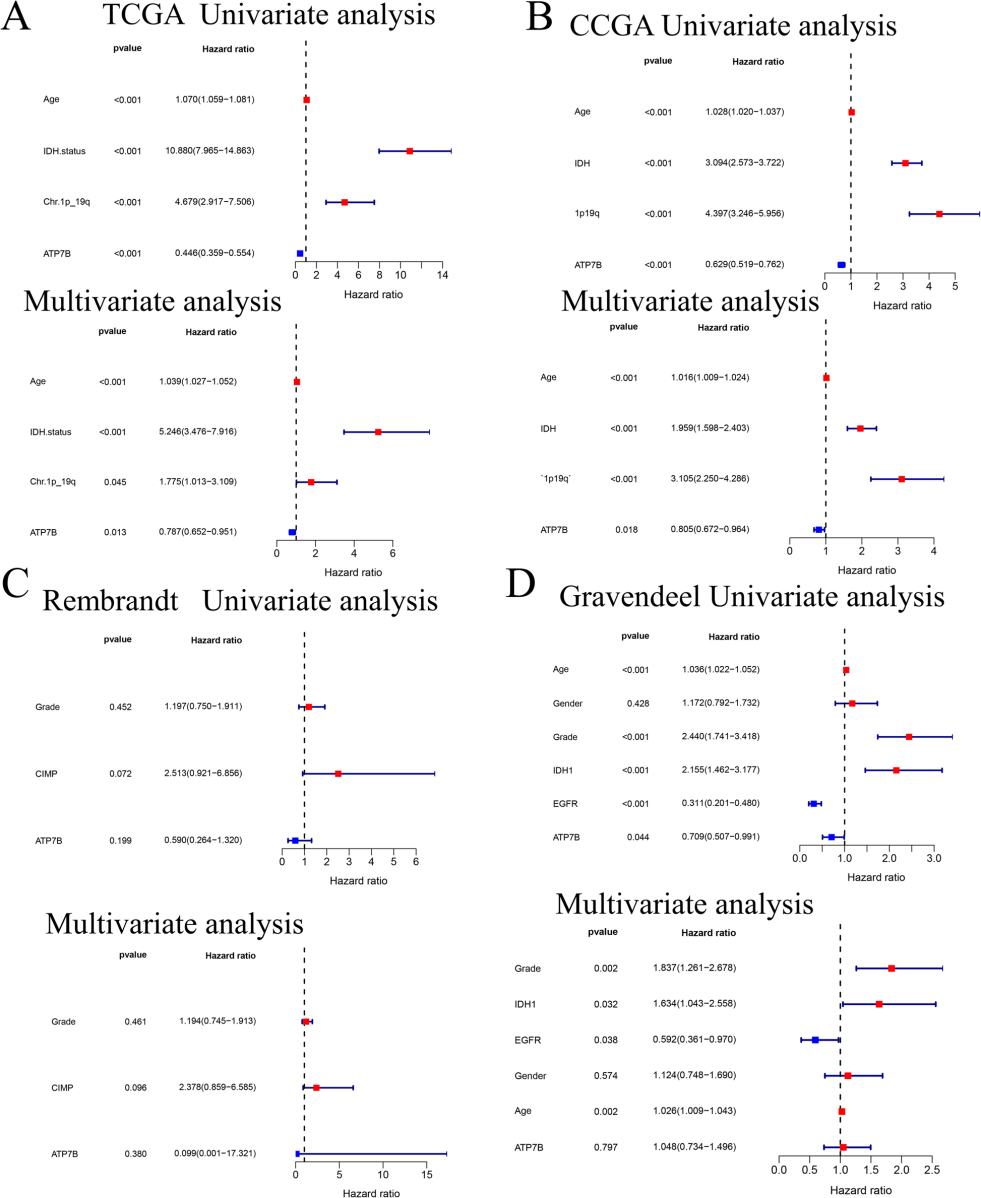
Clinical value of risk signatures and independent prognosis analysis. **A**, **B** A forest plot of univariate and multivariate cox regression analyses revealed that ATP7B could serve as an independent prognostic measure in TCGA and CGGA. **C**, **D** ATP7B failed to serve as an independent prognostic measure in Rembrandt and Gravendeel based on forest plots of univariate and multivariate cox regressions

In the TCGA and CGGA database, univariate and multivariate cox regression analyses identified ATP7B as a prognostic factor (TCGA: *P* = 0.013, HR = 0.787, 95% CI: 0.652 − 0.951; CGGA: *P* = 0.018, HR = 0.805, 95% CI: 0.672 − 0.964) (Fig. 5A, B). However, the Gravendeel and Rembrandt database were used to validate the above findings, which were not statistically significant (Gravendeel: *P* = 0.797, HR = 1.048, 95% CI: 0.734 − 1.496; Rembrandt *P* = 0.380, HR = 0.099, 95% CI: 0.001 − 17.321) (Fig. 5C, D). In different databases, the evaluation of the prognostic value of ATP7B in glioma patients was inconsistent, indicating that ATP7B had greater research space and was more worthy of study.

### Developing and validating a predictive nomogram

**Fig. 6:**
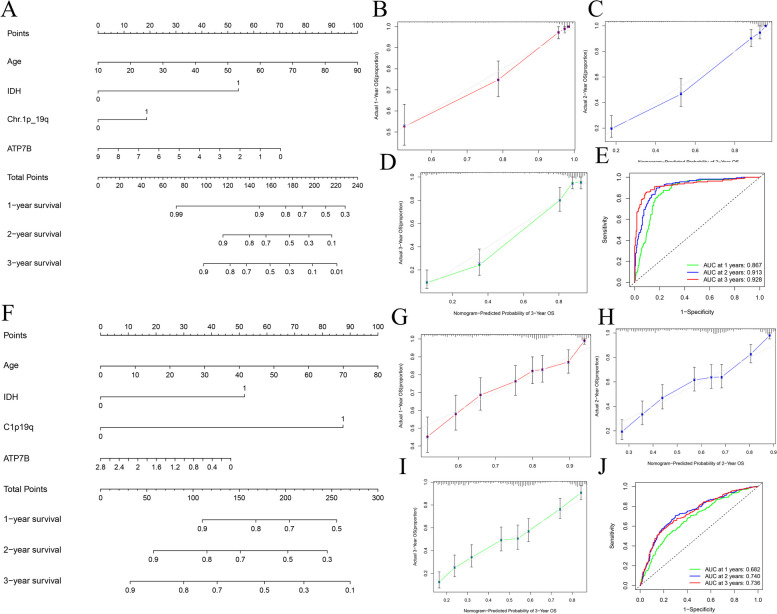
Nomogram construction and validation for gliomas. **A** TCGA-based glioma prognostic nomogram for overall survival of 1 year, 2 years and 3 years. **B-D** A 1-year, a 2-year and a 3-year nomogram calibration curve predicting overall survival in TCGA. **E** The ROC curves for 1-, 2-, and 3-year predictions of TCGA overall survival. **F** CGGA-based glioma prognostic nomogram for OS of 1 year, 2 years and 3 years. **G-I** A 1-year, a 2-year and a 3-year nomogram calibration curve predicting overall survival in CGGA. **J** The ROC curves for 1-, 2-, and 3-year predictions of CGGA overall survival. (The grey line indicates perfect prediction, and the red, blue and green lines represent the predictive ability of the nomogram, respectively. The higher the fit between the red line, blue line, and green line and the gray line, the better the predictive ability of the prediction model.)

We developed a nomogram for OS by quantifying the prognostic model to comprehensively understand the value of some prognostic factors on patient survival, such as Age, IDH, Chr.1p_19q, and ATP7B in the TCGA cohort and Age, IDH, C1p19q, and ATP7B in the CGGA cohort (Fig. 6A, F). As a result of calculating TCGA and CGGA data, the calibration curves showed good consistency when comparing predicted and observed OS results in 1 year, 2 years, and 3 years (Fig. 6B-D, G- I). ROC curves were used to further quantify the difference between the curves to investigate how well the nomogram predicted. In TCGA, the area under the roc curve was 0.866, 2 years was 0.91, and 3 years was 0.93, respectively; in CGGA, the area under the roc curve was 0.682, 2 years was 0.740, and 3 years was 0.736, respectively (Fig. 6E, J).

### ATP7B expression in cancerous and paracancerous tissues

**Fig. 7:**
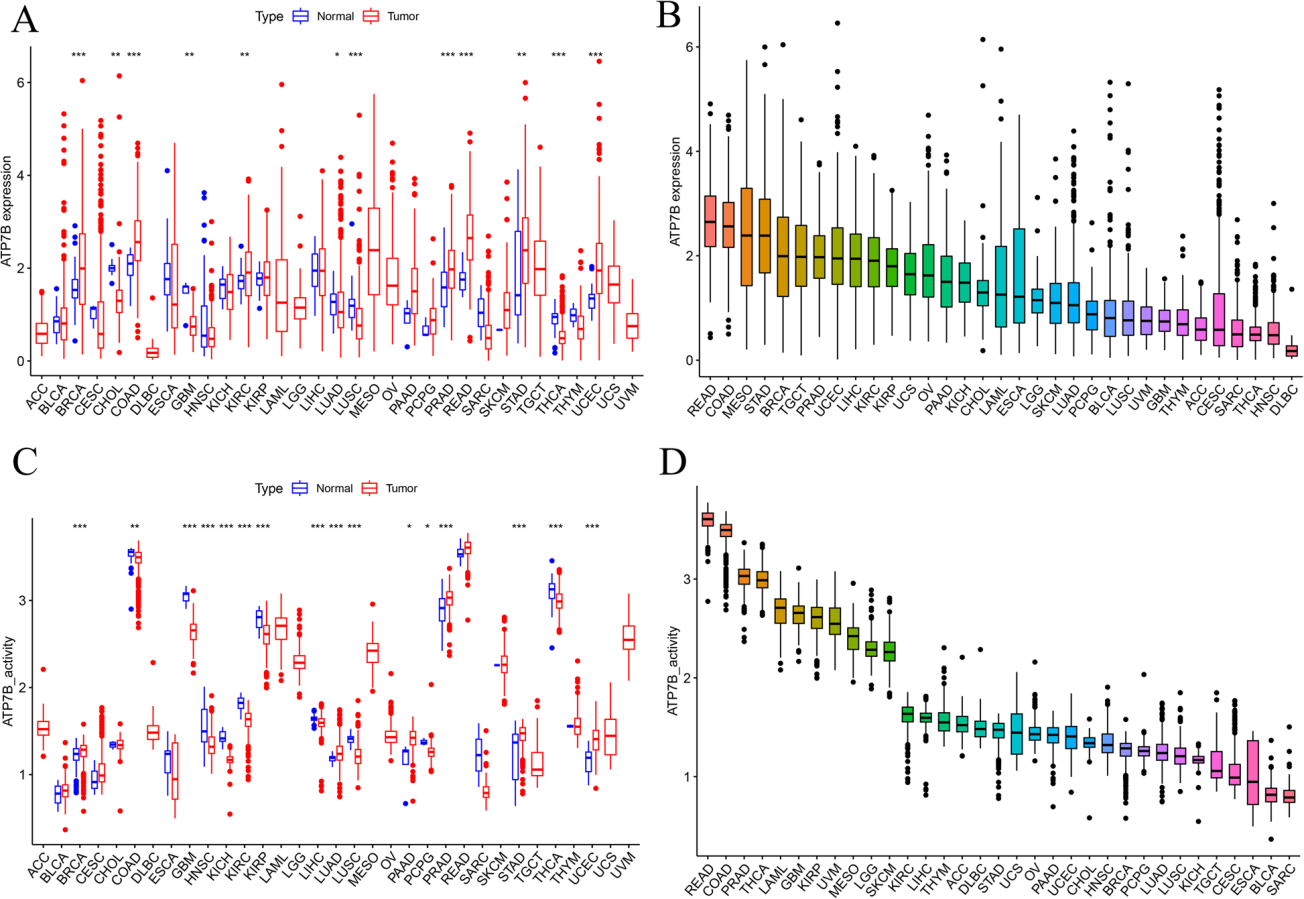
Analyses of ATP7B activity levels and expression across cancer types. **A** The expression levels of ATP7B in 12 out of 24 types of human cancers varied compared to healthy tissues. **B** 33 hunman cancer tissues with ATP7B expression levels. **C** Cancers and healthy tissues with different levels of ATP7B activity. **D** 33 human cancer tissues with different levels of ATP7B activity. (*p < 0.05, **p < 0.01, ***p < 0.001)

The expression of ATP7B varied significantly in 12/24 cancer types, including BRCA, COAD, CHOL, GBM, KIRC, LUAD, LUSC, PRAD, READ, STAD, THCA, UCEC (Fig. 7A). Cancer cells expressed significantly more ATP7B than non-tumor cells in seven types of cancer: BRCA, COAD, KIRC, PRAD, READ, STAD, UCEC. The TIMER database confirmed this result (Supplementary Fig. 1). Subsequently, we described a sorting box plot showing the expression levels of ATP7B in 33 different types of cancer: highest expression in READ and lowest expression in DLBC(Fig. 7B). By analyzing the TCGA database, we determined that there were significant differences in the activity of ATP7B between 16 types of cancer tissues and normal tissues, including BRCA, COAD, GBM, HNSC, KICH, KIRC, KIRP, LIHC, LUAD, LUSC, PAAD, PCPG, PRAD, STAD, THCA, UCEC (Fig. 7C). We also described a sorting box plot showing the activity levels of ATP7B in 33 different types of cancer: highest activity in READ and lowest activity in SARC (Fig. 7D).

**Fig. 8:**
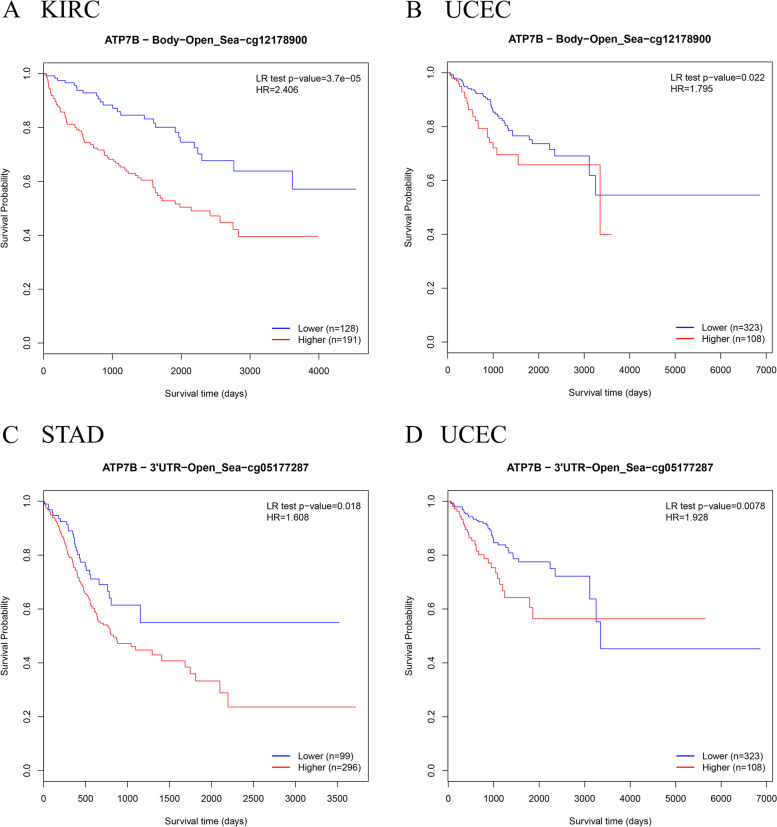
ATP7B methylation and cancer prognosis. **A**, **B** Higher ATP7B − Body − Open_Sea − cg12178900 methylation levels reduced survival in KIRC and UCEC patients. **C**, **D** Higher ATP7B − 3'UTR − Open_Sea − cg05177287 methylation levels reduced survival in STAD and UCEC patients

We performed MethSurv for multivariable survival analysis and evaluated the prognostic potential of DNA methylation biomarkers. Specifically, high methylation of ATP7B − Body − Open_Sea − cg12178900 site (KIRC:*P* = 3.7e-05, HR = 2.406, UCEC: *P* = 0.022, HR = 1.795) and ATP7B − 3'UTR − Open_Sea − cg05177287 site (STAD:*P* = 0.018, HR = 1.608, UCEC: *P* = 0.0078, HR = 1.928) represented poor OS (Fig. 8A-D).

### Patient gliomas and cells expressing ATP7B

**Fig. 9:**
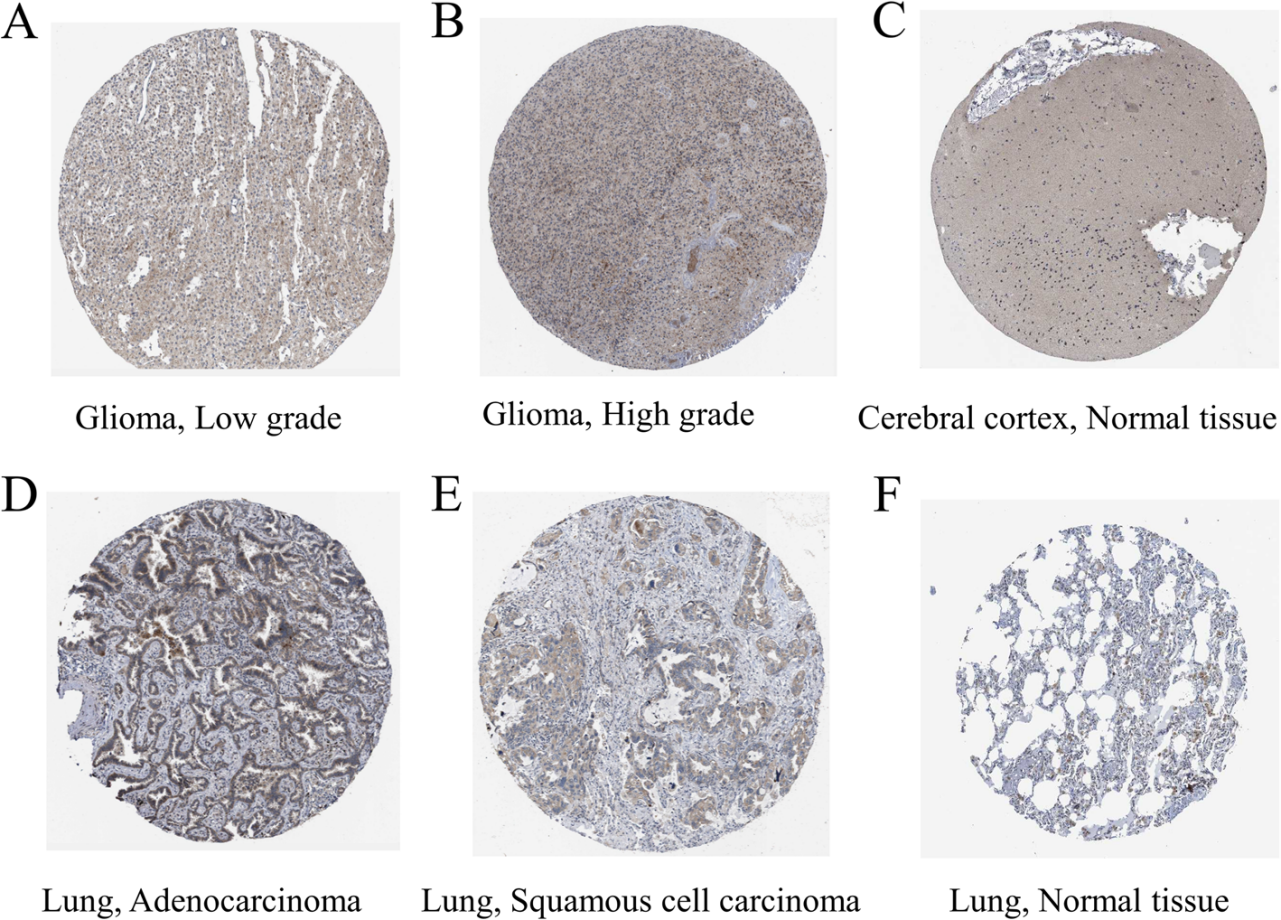
A comparison of ATP7B gene expression in tumors and normal tissues. **A**-**F** An immunohistochemistry study of ATP7B gene expression in normal and tumorous lung and brain tissues

**Fig. 10:**
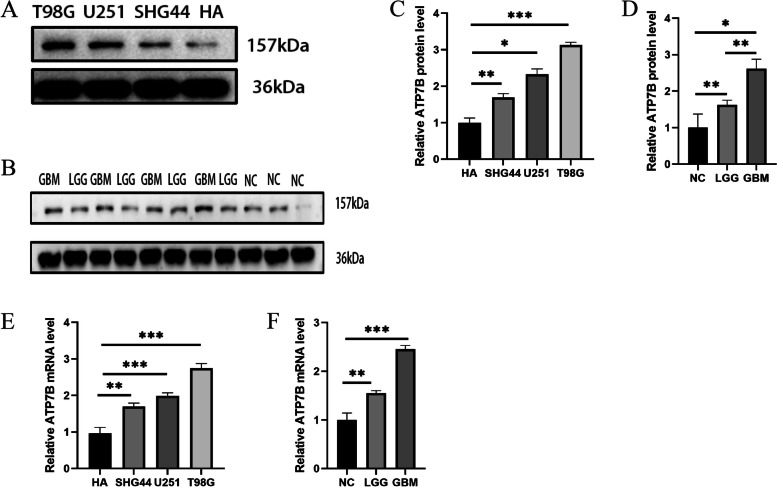
Expression of ATP7B in HA cell lines and glioma cell lines and in normal and glioma tissues. **A**, **C** Representative blot and summary data showed ATP7B protein levels in HA cell lines and glioma cell lines (*n* = 16). **B**, **D** Representative blotting and summary data showed ATP7B protein levels in normal and glioma tissues (*n* = 11). **E** The relative mRNA levels of HA cells and glioma cell line ATP7B were analyzed (*n* = 16). **F** The relative mRNA levels of ATP7B in normal and glioma tissues were analyzed (*n* = 11). (The experiment was independently repeated three times. The data were expressed as mean ± SD, and the student t test was used to compare the values between groups. *p < 0.05, **p < 0.01, ***p < 0.001.)

The HPA database contains millions of immunohistochemical images that we used to compare ATP7B protein expression between normal and tumor tissues. These images were extracted from immunohistochemical examinations of lung and brain tissues (Fig. 9A-F). Compared with normal group, the expression of ATP7B was up-regulated in glioma group, and the up-regulation was more obvious in high-grade gliomas than in low-grade gliomas (Fig. 10A-F, Supplementary Fig. 3A-B).

### cBioPortal Database and GeneMANIA analysis

**Fig. 11:**
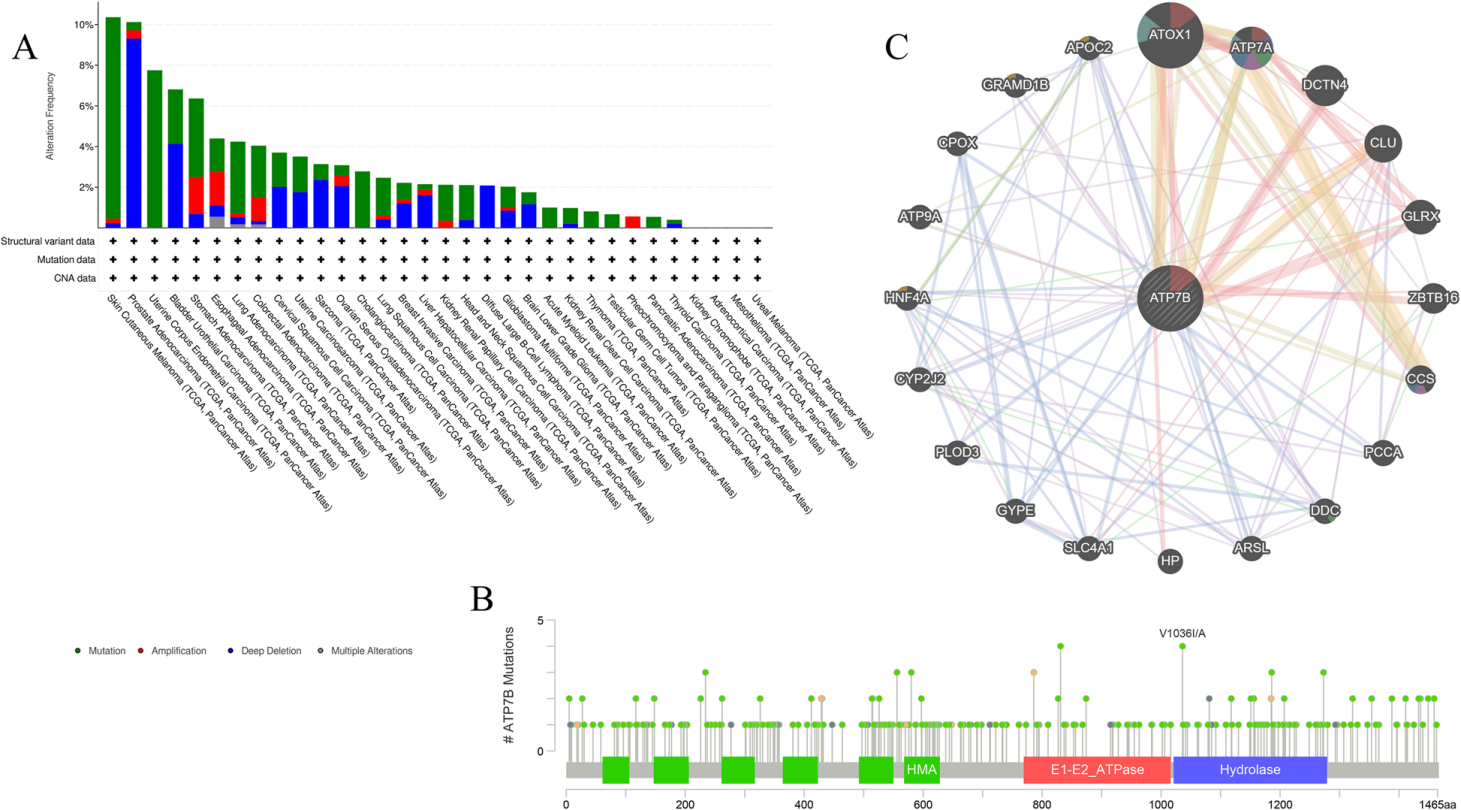
A web tool to analyze mutation characteristics and protein–protein interactions (PPIs) of ATP7B. **A** Data from cBioPortal was used to quantitate ATP7B mutation frequency. **B** The mutation site of ATP7B was obtained from the cBioPortal database. **C** PPI network of ATP7B generated using GeneMANIA. (Pink represents Physical Interactions; Purple represents Co-expression; Orange represents Predicted, blue represents Co-localization; Green represents Genetic Interactions; Light blue represents the Pathway; Gold represents Shared protein domains.)

The cBio Cancer Genomics Portal (cBioPortal) database was used to examine 32 different TCGA cancer studies for mutations in ATP7B. Mutations were the most prevalent alterations in the ATP7B gene among cancers (up to 9.91% of samples in SKCM) (Fig. 11A). In PRAD, 9.31% of the ATP7B gene had a deep deletion as the most common alteration. Additionally, we also annotated genetic alterations of ATP7B sequence, which included location and type information. The primary type of alteration was ATP7B missense mutation, and Chromo V1036I/A alteration was detected in COAD, HNSC, and UCEC (Fig. 11B).

A network of protein interactions between ATP7B and other proteins was created using the GeneMANIA online tool to investigate cancer development and occurrence. In the network, genes exhibited co-expression, physical and genetic interactions, pathway, co-localization, shared protein domains, and predicted interactions. ATOX1 and ATP7B were analyzed based on their predicted, physical interactions, and shared protein domain network using GeneMANIA. A strong physical interaction was present between ATP7B and ATOX1, DCTN4, and GLRX (Fig. 11C).

### Analysis of ATP7B survival and prognosis in pan-cancer patients

**Fig. 12:**
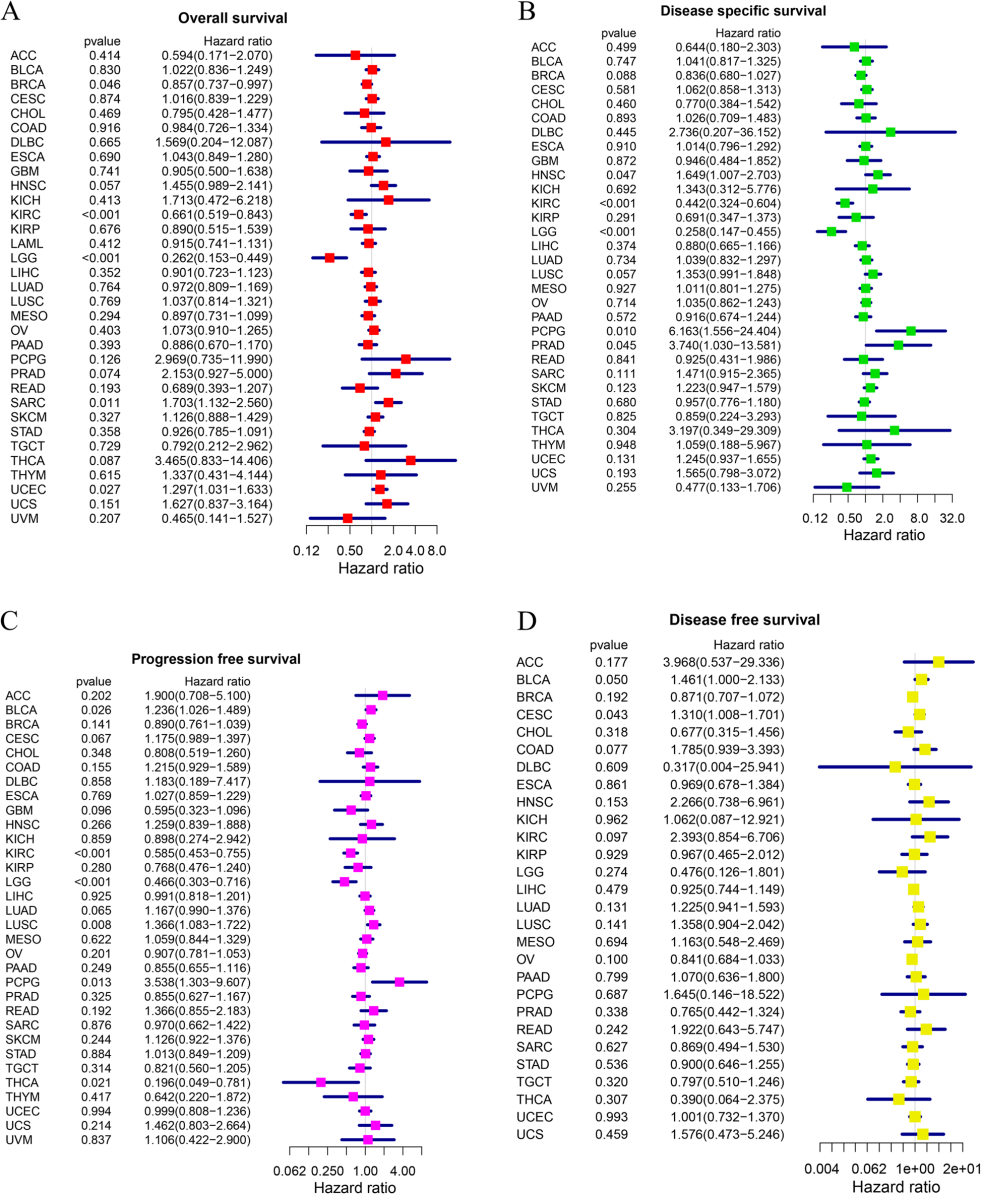
Pan-cancer ATP7B mRNA expression and prognosis. The forest plot illustrates the connections between the expression of ATP7B and the survival rates of four different types (**A** Overall survival, OS; **B** Disease specific survival, DSS; **C** Progression free survival, PFS; **D** Disease free survival, DFS)

**Fig. 13:**
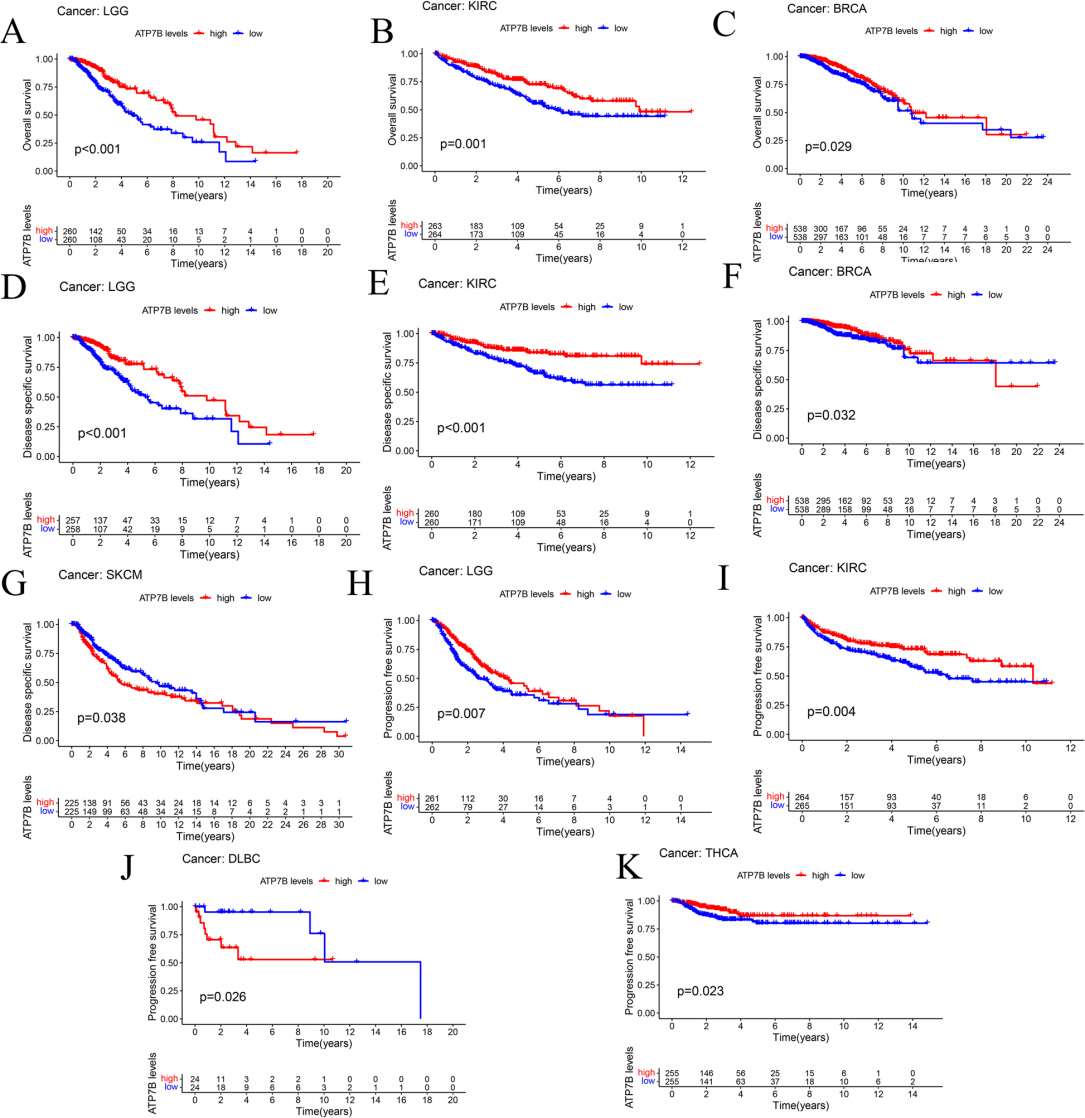
The predictive ability of the ATP7B expression was assessed by K-M survival analysis in various cancers. **A**-**C** Overall survival of BRCA, KIRC, and LGG patients. **D**-**G**. Disease specific survival of KIRC, LGG, BRCA, and SKCM patients. **H**–**K** Progression free survival of THCA, KIRC, LGG, and DLBC patients. (Disease specific survival, DSS; Disease free survival, DFS; Overall survival, OS; Progression free survival, PFS; Kaplan–Meier survival curves depict blue as low expression and red as high expression.)

ATP7B expression levels and survival were analyzed using K-M and cox proportional hazards analysis to learn more about its prognostic value. In this study, we offered four types of survival: Disease specific survival (DSS), Disease free survival (DFS), Overall survival (OS), and Progression free survival (PFS). According to univariate cox regression analysis, the levels of the transcript ATP7B correlated significantly with prognosis in BRCA (*p* = 0.046), KIRC (*p* < 0.001), LGG (*p* < 0.001), SARC (*p* = 0.011), UCEC (*p* = 0.027) (Fig. 12A). While ATP7B had the negative correlation with BRCA, KIRC, and LGG, a positive correlation existed between ATP7B and SARC and UCEC (Fig. 12A). ATP7B gene expression levels were examined as predictive factors for 33 cancer types using K-M survival analyses. The group with high ATP7B expression had a better prognosis than those with low expression in BRCA (*p* = 0.029), KIRC(*p* = 0.001), and LGG(*p* < 0.001) (Fig. 13A-C).

Afterwards, we examined how ATP7B expression was related to DSS. A significant negative association existed between the expression of ATP7B and the HNSC(*p* = 0.047), PCPG(*p* = 0.010), PRAD(*p* = 0.045) in DSS, whereas KIRC(*p* < 0.001) and LGG (*p* < 0.001) were positively correlated (Fig. 12B). LGG(*p* < 0.001), KIRC(*p* < 0.001), and BRCA(*p* = 0.032) patients with high levels of ATP7B mRNA expression had better prognoses with K-M survival analysis (Fig. 13D-F). A key point to note was the variability that ATP7B mRNA expression was inverse for DSS based on these data in SKCM(*p* = 0.038) (Fig. 13G).

The forest plot of PFS revealed different prognostic values in 6 cancer types: BLCA(*p* = 0.026), LUSC(*p* = 0.008), PCPG (*p* = 0.013), KIRC(*p* < 0.001), LGG(*p* < 0.001), and THCA(*p* = 0.021) (Fig. 12C). K-M curves showed that higher expression levels of ATP7B were associated with better prognoses for THCA (*p* = 0.023), KIRC (*p* = 0.004), LGG (*p* = 0.007) patients and poorer prognosis in DLBC patients (*p* = 0.026) (Fig. 13H-K).

As well, univariate cox regression analysis revealed that ATP7B significantly influenced DFS in CESC (*p* = 0.043) (Fig. 12D).

### Pan-cancer gene set enrichment analysis of ATP7B

**Fig. 14:**
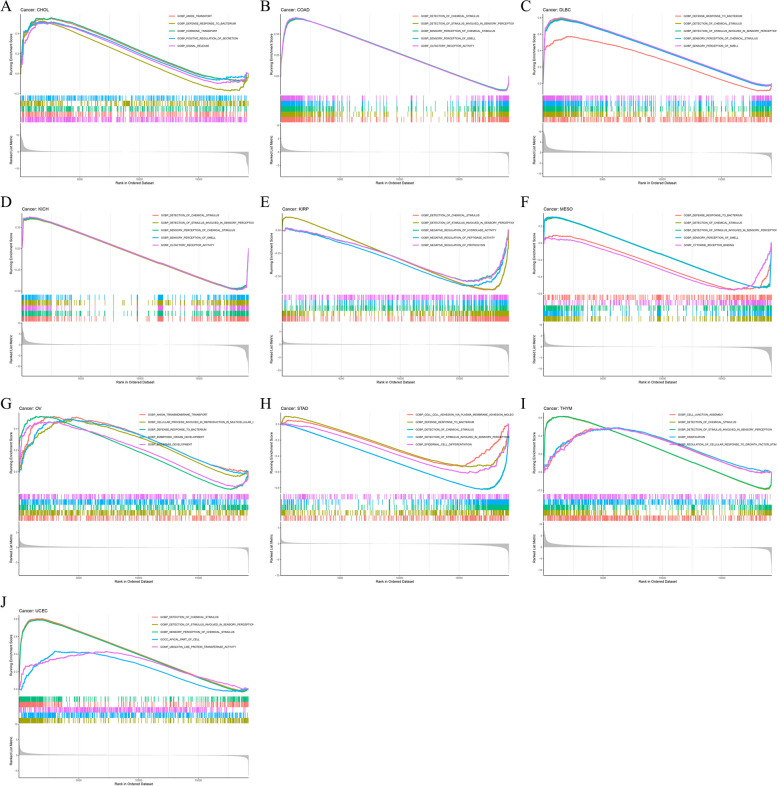
Gene ontology analysis for biological process (BP), cellular component (CC), and molecular function (MF) via Gene Set Enrichment Analysis. **A**-**J** Biological processes of differential expression of ATP7B in different tumor tissues evaluated by Gene Set Enrichment Analysis

We evaluated the biological significance of differentially expressed ATP7B markers using GESA analysis in various tumor tissues. An analysis of GO functional annotations was carried out for biological process (BP), cellular component (CC), and molecular function (MF) in Fig. 14A-J. We found ATP7B protein expression in COAD, DLBC, THYM and UCEC, positively correlated with detection of chemical stimulus and detection of stimulus involved in sensory perception, while in COAD, KIRP, KICH, MESO and STAD negatively correlated with them (Fig. 14B, C, D, E, F, H, I, J). We also analyzed that high expression of ATP7B might participate in the biological process of defense response to bacterium in CHOL, DLBC, and OV (Fig. 14A, C, G). From this, we deduce that ATP7B participates in tumor development and tumorigenesis.

**Fig. 15:**
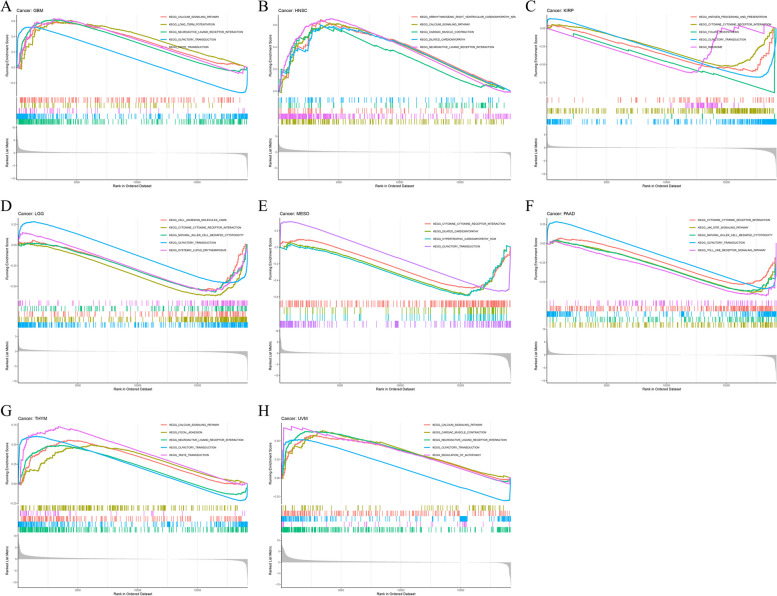
Kyoto Encyclopedia of Genes and Genomes analysis KEGG enrichment analysis. **A**-**H** Kyoto Encyclopedia of Genes and Genomes pathway in differential expression of ATP7B

Subsequently, we analyzed which signaling pathways were enriched in some types of cancer using the KEGG database (Fig. 15A-H). A KEGG enrichment analysis demonstrated that ATP7B had a positive effect on neuroactive ligand receptor interaction and calcium signaling pathway in GBM, HNSC, THYM, and UVM (Fig. 15A, B, E, F). ATP7B negatively regulated cytokine-cytokine receptor interaction and olfactory transduction in KIRP, LGG, MESO, and PAAD (Fig. 15C, D, G, H).

### Tumors with various immune characteristics associated with ATP7B expression

**Fig. 16:**
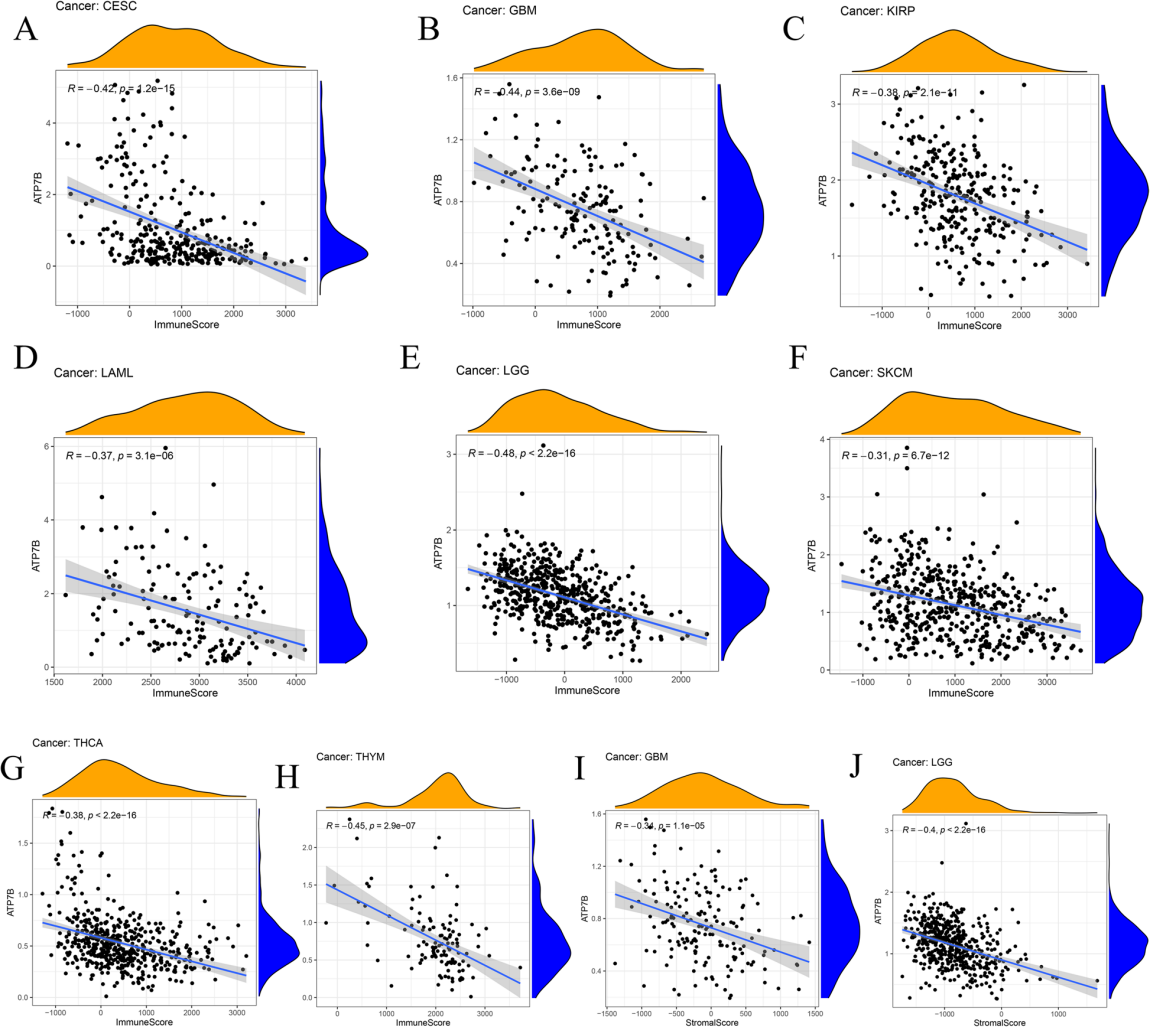
Correlation between immune score and stromal score and ATP7B expression. **A**-**H** Analysis of the negative correlation between immune score and expression of ATP7B. **I**-**J**. Analysis of the negative correlation between stromal score and expression of ATP7B

A model of estimate score, stromal score, immune score, and tumor purity was created using the ESTIMATE tool. The immune score indicated immune cells infiltrated, while stromal score indicated stromal cells infiltrated. Therefore, the tumor microenvironment (TME) was quantified as stromal cell (stromal score) and immune cell (immune score) scores. The immune score and ATP7B expression were negatively correlated in Fig. 16A-H (*p* < 0.05), including CESC (*R* =—0.42, *p* = 1.2e − 15), GBM(R =—0.44, *p* = 3.6e − 09), KIRP(*R* =−0.38, *p* = 2.1e − 11), LAML(*R* =−0.37, *p* = 3.1e − 06), LGG(*R* =−0.48, *p* < 2.2e − 16), SKCM(*R* =−0.31, *p* = 6.7e − 12), THCA ( *R* =−0.38, *p* < 2.2e − 1), THYM (*R* =−0.45, *p* = 2.9e − 07). ATP7B expression and stromal score were also negatively correlated in GBM (*R* =  − 0.34, *p* = 1.1e − 05) and LGG (*R* =  − 0.4, *p* < 2.2e − 16) (Fig. 16I-J).

We wondered whether immune infiltration occurred in pan-cancer in relation to ATP7B using the CIBERSORT algorithm. A total of eight types of cancer were found to have significant differences (BRCA, ESCA, KIRP, LAML, MESO, TGCT, THYM, UCS) (Supplementary Fig. 2A-Q). For example, ATP7B correlated positively with B cells naive and Mast cells resting, while ATP7B negatively correlated with Macrophages M2 and Monocytes in LAML (Supplementary Fig. 2H-K). Macrophages M2 and NK cells activated, and ATP7B expression exhibited strong correlations in TGCT, as did T cells CD4 memory resting (Supplementary Fig. 2M-O). ATP7B expression and T cells CD4 memory resting showed a positive correlation in THYM, TGCT, and UCS (Supplementary Fig. 2O-Q).

**Fig. 17:**
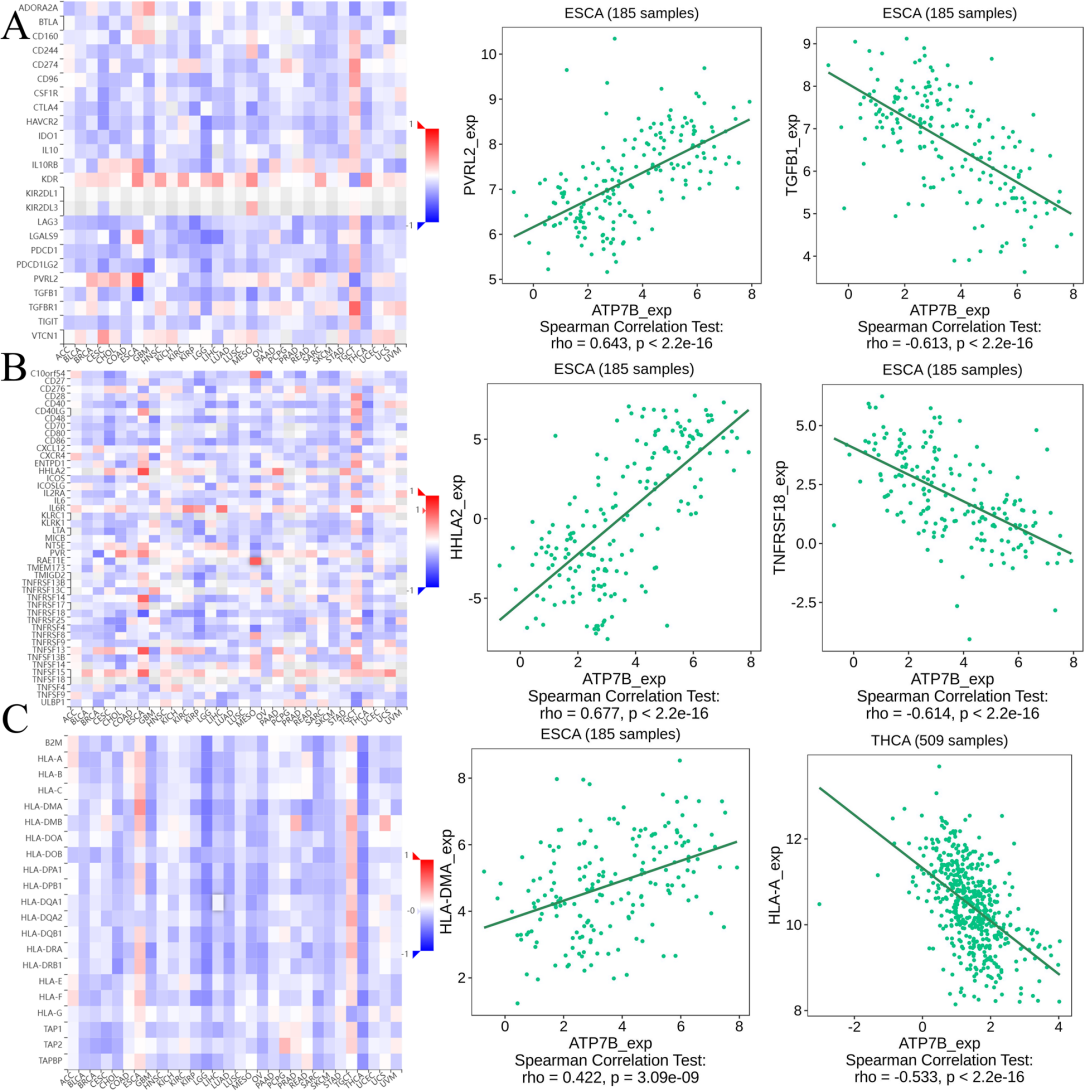
Relations between three kinds of immunomodulators and expression of ATP7B. **A** Correlations between immunoinhibitor and ATP7B and the two immunoinhibitors with the highest correlation values. **B** Correlations between immunostimulator and ATP7B and the two immunostimulators with the highest correlation values. **C** Correlations between MHC molecule and ATP7B and the two MHC molecules with the highest correlation values

Based on a spearman correlation analysis of the TISIDB database, we investigated the relationship between ATP7B expression and immunomodulators in 30 human cancer types, including immunoinhibitory, immunostimulatory, and MHC molecule. There were two immunoinhibitors (PVRL2:rho = 0.643, *p* < 2.2e-16 in ESCA; TGFB1:rho = -0.613, *p* < 2.2e-16 in ESCA) with the highest correlation value with the expression of ATP7B (Fig. 17A). Based on an analysis of 30 human cancers, ATP7B expression was related to immunostimulatory activity, including ESCA(HHLA2:rho = 0.677, *p* < 2.2e-16; TNFRSF18:rho = -0.614, *p* < 2.2e-16) ( Fig. 17B). Based on an analysis of 30 human cancers, ATP7B expression was related to MHC molecule, including ESCA (HLA-DMA2:rho = 0.422, *p* < 3.09e-09) and THCA(HLA-A: rho = -0.533, *p* < 2.2e-16) (Fig. 17C).

### An analysis of Pan-Cancer ATP7B and TMB/MSI associations

**Fig. 18:**
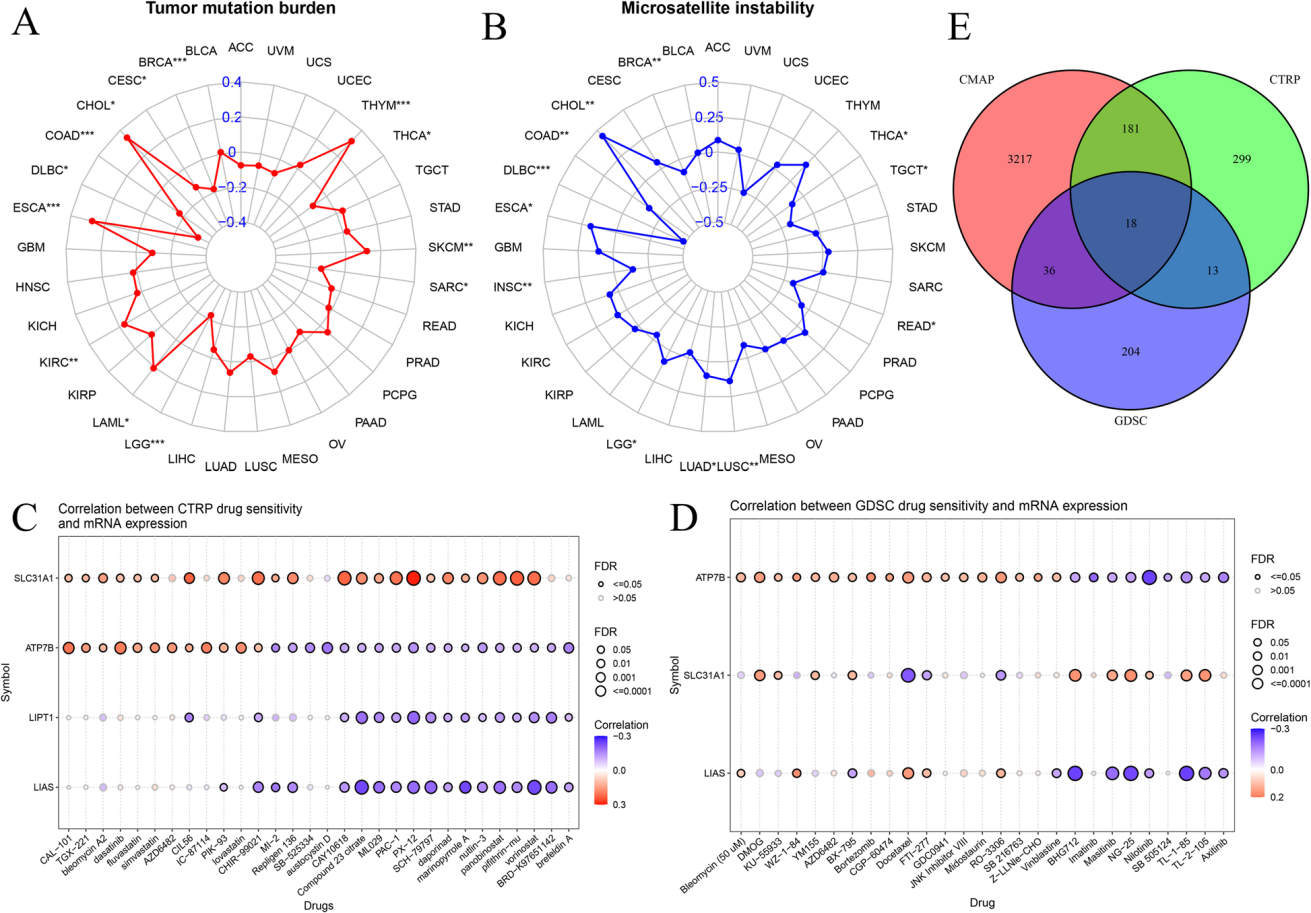
Relationship between TMB, MSI, drug sensitivity, and ATP7B expression in pan-cancers analysis. **A**, **B** Relationship between TMB, MSI and the expression of ATP7B; *p < 0.05, **p < 0.01, ***p < 0.001. **C**, **D** Relationship between CTRP and GDSC drug sensitivity and cuprotosis genes expression in pan-cancer. **E** A Venn diagram with 18 common sensitive drugs

MSI and TMB are significantly correlated with cancer immunotherapy and prognosis. A correlation was observed between TMB or MSI and ATP7B expression in 33 common cancers to assess whether mutations affected the activity of ATP7B (Fig. 18A-B). TMB and ATP7B expression showed a significant positive correlation in CHOL, ESCA, KIRC, LAML, SKCM, THYM, and a negative correlation in BRCA, CESC, COAD, DLBC, LGG, SARC, THCA (*P* < 0.05 is statistically significant) (Fig. 18A). We also found that ATP7B and MSI showed significant positive correlations in 5 of 33 common cancers, including CHOL, ESCA, LGG, LUAD, LUSC, while ATP7B showed significant negative correlations with MSI in 7 of 33 cancers, including BRCA, COAD, DLBC, HNSC, READ, TGCT, THCA (Fig. 18B). Supplementary Table 1 contained a summary of the results.

### Drug susceptibility analysis

As part of our analysis, we examined the correlation coefficients between drug sensitivity and cuprotosis genes using two publicly available large-scale pharmacogenomics resources, CTRP and GDSC. Results that were significant were presented. Two figures (Fig. 18C and D) illustrated separately how drug sensitivity (CTRP and GDSC) correlated with RNA expression in pan-cancers. There was a positive correlation between SLC31A1 expression and 23 kinds of compounds in the study, while LIPT1 and LIAS had a negative correlation with many anticancer drugs (Fig. 18C). The expression of cuprotosis-related genes (ATP7B, SLC31A1and LIAS) was associated with resistance to multiple antitumor drugs (Fig. 18D). Additionally, we found that there were significant differences in the expression of ATP7B and sensitivity to different drugs, with a positive or negative correlation (Fig. 18C-D). Finally, we integrated sensitive drugs with a *P*-value less than 0.05 from three pharmacogenomic resources, CTRP, GDSC, and CMap, and identified 18 drugs associated with cuprotosis-related genes (ATP7B, SLC31A1, and LIAS) (Supplementary table 2). Cross area refered to drugs that were sensitive in all pharmacogenomics databases (Fig. 18E).

## Discussion

An increased interest has been shown in cancer immunometabolism in recent years, which may be a promising method for treating cancer and is essential to improve cancer patient survival. Proliferation, tumor growth, and angiogenesis are all dependent on copper [32]. Previous studies have shown that many cancers exhibit platinum resistance due to ATP7B [9, 12, 13, 33, 34]. The ATP7B system that is one of the copper transporter components, is still being explored in the field of cancer. As the first comprehensive study to examine ATP7B expression and immune checkpoint genes as well as clinical prognosis, mutations, and immune cell infiltration, we hope to make certain contributions to this field.

A comprehensive examination of ATP7B expression in various tumor types was conducted in our study. This study revealed that ATP7B was significantly overexpressed in 7 tumors (BRCA, COAD, KIRC, PRAD, READ, STAD, UCEC) compared with normal tissues, and lowly expressed in 5 tumors (CHOL, GBM, LUAD, LUSC, THCA). Additionally, the abnormal expression of ATP7B was linked to prognosis (DFI, PFI, DSS, and OS) in a variety of tumors, which might have potential prognostic implications for patients with LGG and KIRC. Kaplan–Meier survival data showed that ATP7B expression was associated with a favorable prognosis for BRCA, KIRC, and LGG. In our study, ATP7B expression in gliomas was higher than in normal tissues, and it was significantly higher in high-grade gliomas than in low-grade gliomas.

TMB detects cancer cells by measuring the number of mutations in them. Tumors undergo microsatellite instability as a result of insertions or deletions of repeat units, leading to changes in microsatellite length and the appearance of new alleles of microsatellite. Studies have shown that MSI and TMB may be immune therapy response biomarkers [15, 16]. This study shows that ATP7B is also significantly correlated with MSI and TMB. Therefore, we speculate that ATP7B is associated with immunotherapy for cancers. Gene Network Analysis revealed the physical interactions, predicted, and shared protein domains network for ATP7B and ATOX1. Copper could be transferred to the secretory pathway by ATOX1. As early as the pre-neoplastic stage, aberrant ATOX1 expression can be detected, which would be useful for detecting tumors early and stratifying patients for adjuvant treatment [35].

ATP7B expression was also examined in 33 human cancers with various immune cell subtypes using the CIBERSORT algorithm to determine its potential mechanism of action. Our research demonstrated that in BRCA, ESCA, KIRP, LAML, MESO, TGCT, THYM, and UCS, a significant correlation was found between multiple immune cells infiltration and ATP7B expression, suggesting that ATP7B might be an immunotherapeutic target. ATP7B expression had significantly negative correlations with immune score and stromal score in GBM and LGG. Our study also found that the expression of ATP7B was significantly correlated with T cells regulatory (T regs), Mast cells resting, Macrophages M2, and B cells naive in LAML. Boutilier AJ, et al. claimed that tumor-associated macrophages (TAMs), commonly known as m2-polarized macrophages, contributed to the development of tumors and played a vital role in angiogenic and lymphangiogenic regulation, immune suppression, hypoxia induction, tumor cell proliferation, and metastasis [36]. TAMs were the main components of the TME and were often associated with poor prognosis and immunotherapy resistance (including immunotherapy), especially in advanced diseases. Therefore, targeting TAMs may have therapeutic potential in tumor immunotherapy, which brings hope for successful cancer treatment [37]. Lipid-loaded TAMs released CCL6 to encourage cancer cell proliferation and migration. The Marco interference inhibited tumor growth by targeting lipid accumulation and invasiveness and improved chemotherapy efficacy in models of prostatic adenocarcinoma [38].

GSEA was performed to identify ATP7B with biological significance in pan-cancer. GO enrichment analysis showed high expression of ATP7B participated in the biological process of defense response to bacterium. KEGG enrichment analysis showed that ATP7B negatively regulated cytokine-cytokine receptor interaction. Cu dysregulation might participate in tumor metastasis by inducing inflammatory responses [39]. As a result, we will be able to develop a novel anti-tumor treatment program for cancer by clarifying the mechanism of ATP7B in inflammatory-related activities.

Some study evidences indicated that the CMap was used to discover functional connections between genes, drugs, and disease states, predicting the potential effects of drugs on cancers [18, 19, 40]. This study ultimately screened 18 small molecule drugs that were sensitive to cancers. Selemetinib, as a selective MEK1 inhibitor for the treatment of solid tumors, could block the mitogen-activated protein kinase/extracellular signal-regulated kinase signaling pathway, thereby regulating cell critical response, and had good activity in pediatric low-grade glioma, non-small cell lung cancer, and melanoma [41]. By activating the p38/MAPK pathway and inhibiting TRIM14 signaling, piperlongumine inhibited GBM growth [42]. SN-38, a CPT analogue, inhibited homologous recombination repair in BRCA-proficient ovarian cancer cells to make them susceptible to PARP inhibitor therapy [43]. Cancer immunotherapies like checkpoint inhibitors and adoptive cell therapy could stimulate the immune system to fight cancer [44]. This study clearly indicates that ATP7B could be a biomarker for the prognosis and diagnosis of various cancers, which is very important for developing precise cancer treatment in the future.

As a result of the limitations of the study, the following conclusions can be drawn. First, this study relied on public online databases and bioinformatics tools, which meant further basic and clinical research was needed to validate its findings. Next, the level of ATP7B expression associates with both immune cell infiltration and cancer patient survival, but we need to clarify the role of ATP7B in immune cell infiltration. Third, our next goal is to test the efficacy of ATP7B in different types of cancer patients, and determines how it works, through in vivo and in vitro studies. Therefore, the discovery of molecular substances that disrupt copper homeostasis may provide a new treatment approach for cancers that rely on copper for growth and angiogenesis.

## Conclusion

It was observed in this study that ATP7B expression was different between tumors and normal tissues and had a correlation with prognosis of patients with tumors. As well, we observed a connection between ATP7B expression and TMB, MSI, and immune infiltration in diverse tumor types. ATP7B is associated with immune infiltration and may serve as a biomarker to provide new insights into the prognosis of pan-cancer patients.

### Supplementary Information

**Additional file 1: Table 1.** Results of MSI and TMB. **Table 2.** Screening out small molecule drugs on CMap website. **Supplementary Figure 1.** Analyses of ATP7B expression levels across cancer types. **Supplementary Figure 2.** Correlation analysis between ATP7B expression and immune infiltration in 8 types of cancer. **Supplementary Figure 3.** Expression of ATP7B in HA cell lines and glioma cell lines and in normal and glioma tissues.

## Data Availability

The datasets generated and analysed during the current study are available in the Cancer Genome Atlas repository, [TCGA, https://portal.gdc.cancer.gov/repository].

## Abbreviations

ACC: Adrenal cortical carcinoma
BLCA: Bladder urothelial carcinoma
BRCA: Breast invasive carcinoma
CESC: Cervical and endocervical cancers
COAD: Colon adenocarcinoma
CHOL: Cholangio carcinoma
DLBC: Lymphoid neoplasm diffuse large B– cell lymphoma
ESCA: Esophageal carcinoma
GBM: Glioblastoma multiforme
HNSC: Head and neck squamous cell carcinoma
KICH: Kidney chromophobe
KIRC: Kidney renal clear cell carcinoma
KIRP: Kidney renal papillary cell carcinoma
LAML: Acute myeloid leukemia
LIHC: Liver hepatocellular carcinoma
LGG: Lower grade glioma
LUAD: Lung adenocarcinoma
LUSC: Lung squamous cell carcinoma
MESO: Mesothelioma
OV: Ovarian serous cystadenocarcinoma
PAAD: Pancreatic adenocarcinoma
PCPG: Pheochromocytoma and paraganglioma
PRAD: Prostate adenocarcinoma
READ: Rectum adenocarcinoma
SARC: Sarcoma
SKCM: Skin cutaneous melanoma
STAD: Stomach adenocarcinoma
TGCT: Testicular germ cell tumors
THCA: Thyroid carcinoma
THYM: Thymoma
UCEC: Uterine corpus endometrial carcinoma
UCS: Uterine carcinosarcoma
UVM: Uveal melanoma
TCA: Tricarboxylic acid
TCGA: The Cancer Genome Atlas
CTRP: Cancer Therapeutics Response Portal
GDSC: Genomics of Drug Sensitivity in Cancer
CMap: Connectivity map
GSCA: Gene Set Cancer Analysis
OS: Overall survival
DSS: Disease-specific survival
DFI: Disease-free interval
PFI: Progression-free interval
TIMER: Tumor Immune Estimation Resource
GSEA: Gene Set Enrichment Analysis
GO: Gene ontology
KEGG: Kyoto Encyclopedia of Genes and Genomes
MSigDB: Molecular Signatures Database
TMB: Tumor mutation burden
MSI: Microsatellite instability
HAs: Human astrocytes
RPMI: Roswell Park Memorial Institute
MEM: Minimal essential medium
FBS: Fetal bovine serum
cBioPortal: CBio Cancer Genomics Portal
BP: Biological process
CC: Cellular component
MF: Molecular function
TME: Tumor microenvironment
T regs: T cells regulatory
TAMs: Tumor-associated macrophages
