# Supplementary material for: An association between ATP7B expression and human cancer prognosis and immunotherapy: a pan-cancer perspective
Source: BMC Med Genomics. 2023 Nov 30;16:307. doi: 10.1186/s12920-023-01714-5 (PMC10687837; doi:10.1186/s12920-023-01714-5)
Supplement: Supplementary file 1 — Additional file 1: Table 1. Results of MSI and TMB. Table 2. Screening out small molecule drugs on CMap website. Supplementary Figure 1. Analyses of ATP7B expression levels across cancer types. Supplementary Figure 2. Correlation analysis between ATP7B expression and immune infiltration in 8 types of cancer. Supplementary Figure 3. Expression of ATP7B in HA cell lines and glioma cell lines and in normal and glioma tissues. [file 12920_2023_1714_MOESM1_ESM.docx]

Table1 Results of MSI and TMB

| CancerType | TMB | | MSI | |
| --- | --- | --- | --- | --- |
|  | cor | pValue | cor | pValue |
| ACC | -0.075936425 | 0.505960265 | 0.085431906 | 0.454096925 |
| BLCA | 0.010065301 | 0.839376956 | 0.008942974 | 0.857083 |
| BRCA | -0.181585432 | 1.03E-08 | -0.096098833 | 0.00194791 |
| CESC | -0.12552872 | 0.033838854 | 0.054628768 | 0.344089148 |
| CHOL | 0.34252236 | 0.040862125 | 0.444272844 | 0.007111524 |
| COAD | -0.169058251 | 0.000730344 | -0.147590125 | 0.00223102 |
| DLBC | -0.330488383 | 0.046281023 | -0.478870947 | 0.000574969 |
| ESCA | 0.274131962 | 0.000451863 | 0.184873236 | 0.019265287 |
| GBM | -0.093257351 | 0.257957776 | 0.103040027 | 0.206502486 |
| HNSC | 0.021950531 | 0.627175343 | -0.136286603 | 0.002351737 |
| KICH | 0.026185985 | 0.835965146 | 0.067052049 | 0.595626996 |
| KIRC | 0.168029172 | 0.002126582 | 0.076767372 | 0.160947718 |
| KIRP | 0.074554331 | 0.215273863 | 0.033242659 | 0.576237629 |
| LAML | 0.206868154 | 0.033365556 | -0.043807611 | 0.644996038 |
| LGG | -0.226491475 | 3.00E-07 | 0.088145637 | 0.047069524 |
| LIHC | -0.049652283 | 0.348206255 | -0.041069925 | 0.431526176 |
| LUAD | 0.062388996 | 0.162377679 | 0.100427718 | 0.023184385 |
| LUSC | -0.030224216 | 0.505337796 | 0.138282901 | 0.002088278 |
| MESO | 0.082236988 | 0.471211357 | -0.095000708 | 0.395890389 |
| OV | -0.001982616 | 0.974035372 | -0.011234783 | 0.853667285 |
| PAAD | -0.056807784 | 0.488419573 | 0.008404768 | 0.91210026 |
| PCPG | 0.054894909 | 0.468021364 | 0.072667789 | 0.335069042 |
| PRAD | -0.021796677 | 0.633113398 | -0.061057188 | 0.175013941 |
| READ | -0.052690286 | 0.54848986 | -0.181112062 | 0.025551141 |
| SARC | -0.136792831 | 0.036111116 | 0.010130868 | 0.87260737 |
| SKCM | 0.120055736 | 0.009562091 | 0.03949876 | 0.393916415 |
| STAD | 0.022952735 | 0.660755977 | -0.030159056 | 0.560951829 |
| TGCT | 0.038135384 | 0.648820024 | -0.182406347 | 0.025476412 |
| THCA | -0.095402153 | 0.036273703 | -0.098375408 | 0.029288118 |
| THYM | 0.315522974 | 0.000529806 | 0.160423081 | 0.082679198 |
| UCEC | 0.025693599 | 0.556929681 | 0.033332886 | 0.440371117 |
| UCS | -0.083944549 | 0.538487111 | -0.254690864 | 0.058183275 |
| UVM | -0.067948382 | 0.549255238 | 0.030312088 | 0.789535484 |

Specific values showing significant correlation between ATP7B and TMB or MSI in common cancers. Statistical significance was determined by a P value < 0.05.

| pert_iname | cell_iname | nsample | raw_cs | norm_cs | Pvalue |
| --- | --- | --- | --- | --- | --- |
| selumetinib | PC3 | 3 | 0.4563 | 1.5648 | 0.010368 |
| BI-2536 | HCT116 | 2 | 0.4407 | 1.5113 | 0.012086 |
| PAC-1 | HA1E | 2 | 0.3834 | 1.3149 | 0.018467 |
| PI-103 | THP1 | 2 | 0.3789 | 1.2993 | 0.022856 |
| OSI-027 | PHH | 4 | 0.3716 | 1.2742 | 0.028728 |
| MK-2206 | NCIH2073 | 2 | 0.363 | 1.2447 | 0.048062 |
| PHA-793887 | HS578T | 3 | -0.3363 | -1.0813 | 0.049283 |
| BMS-536924 | SW480 | 2 | -0.3367 | -1.0826 | 0.049249 |
| SNX-2112 | MCF10A | 2 | -0.3372 | -1.084 | 0.048062 |
| MG-132 | HUVEC | 2 | -0.3373 | -1.0844 | 0.048018 |
| piperlongumine | THP1 | 2 | -0.3405 | -1.0949 | 0.042894 |
| OSI-930 | PC3 | 2 | -0.3407 | -1.0952 | 0.042875 |
| EX-527 | CD34 | 3 | -0.3442 | -1.1066 | 0.038628 |
| BMS-754807 | A375 | 3 | -0.3461 | -1.1127 | 0.037 |
| SN-38 | A375 | 3 | -0.3497 | -1.1242 | 0.030697 |
| CHIR-99021 | NPC | 3 | -0.3517 | -1.1306 | 0.02902 |
| TPCA-1 | NOMO1 | 2 | -0.3519 | -1.1313 | 0.028907 |
| KU-55933 | A375 | 2 | -0.3553 | -1.1423 | 0.02614 |

Table2 Screening out small molecule drugs on CMap website

Statistical significance was determined by a P value < 0.05.


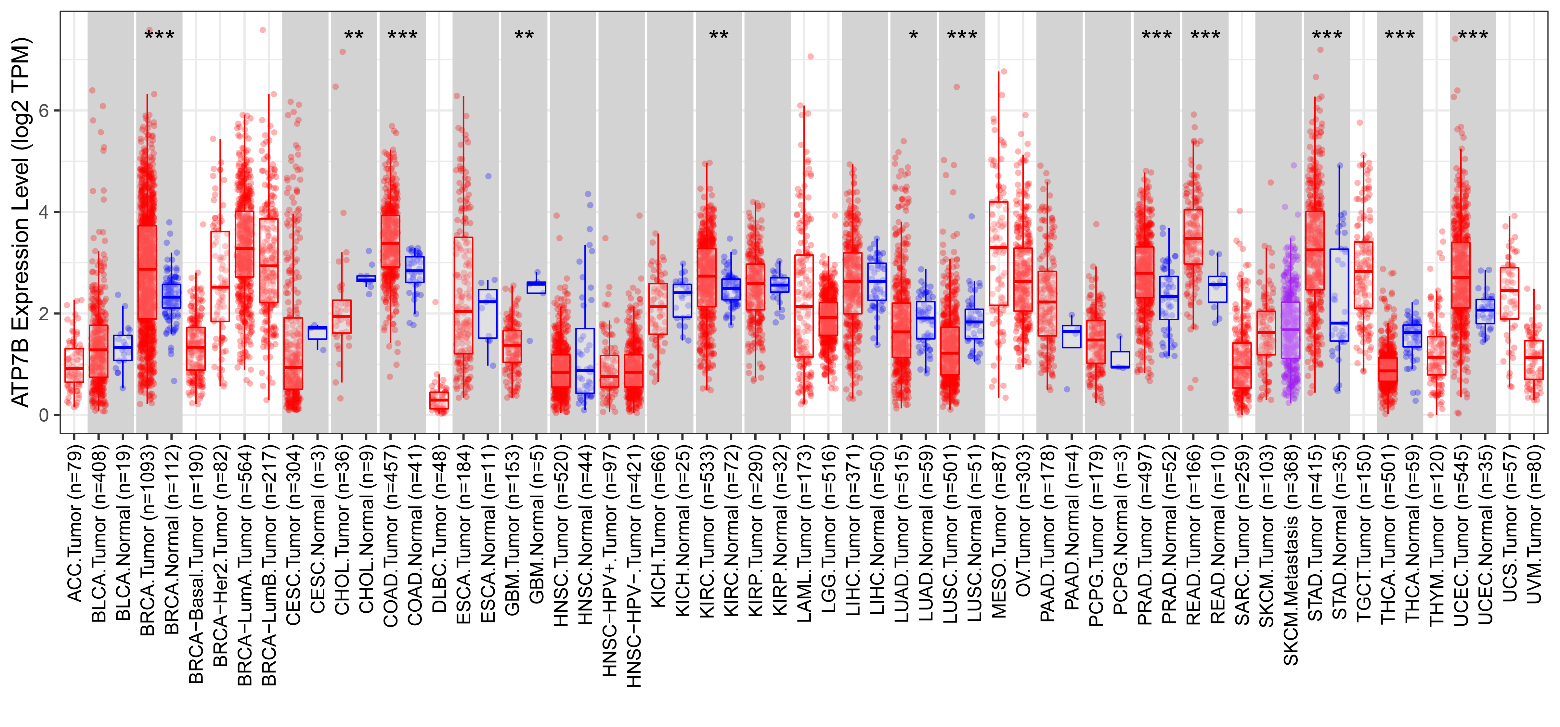


Supplementary Figure1 Analyses of ATP7B expression levels across cancer types

In 7 types of tumors, the expression of ATP7B is higher than that of normal tissue, and in 5 types of cancers, the expression of ATP7B is lower than that of normal tissue.


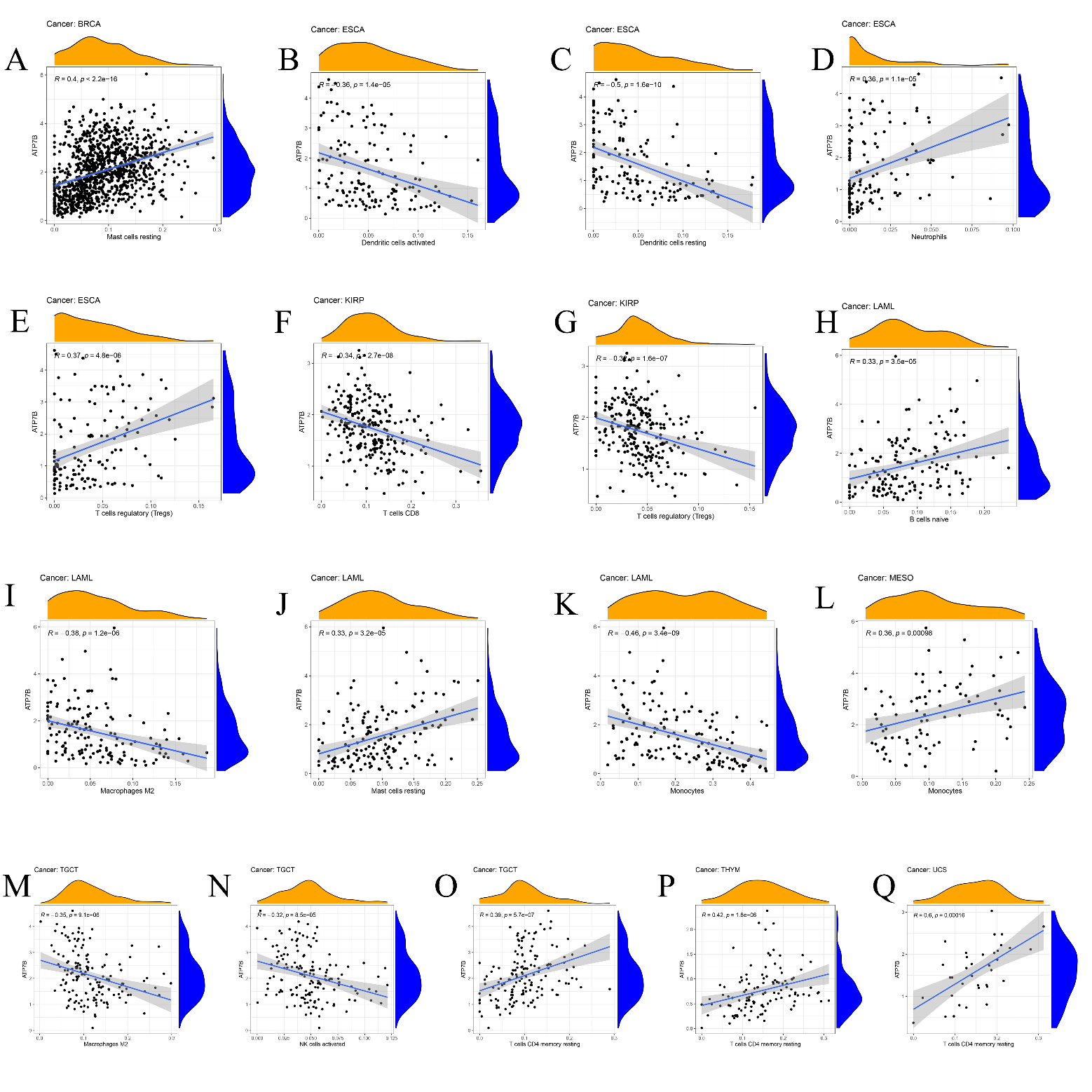


Supplementary Figure2 Correlation analysis between ATP7B expression and immune infiltration in 8 types of cancer

A. A positive correlation was observed between ATP7B expression and Mast cells resting infiltration in BRCA.

B-E. There was a negative correlation between ATP7B expression and Dendritic cells activated and Dendritic cells resting in ESCA tissues, whereas ATP7B expression correlated positively with infiltration of Neutrophils and T cells regulatory (Tregs).

F-G. There was a negative correlation between ATP7B expression and T cells CD8 and T cells regulatory (Tregs) in KIRP tissues.

H-K. ATP7B correlated positively with B cells naive and Mast cells resting, while ATP7B negatively correlated with Macrophages M2 and Monocytes in LAML.

L. There was a negative correlation between ATP7B expression and Monocytes in MESO tissues.

M-O. Macrophages M2 and NK cells activated, and the expression of ATP7B exhibited a strong negative correlation in TGCT, while T cells CD4 memory resting showed a strong positive correlation.

P-Q. ATP7B expression and T cells CD4 memory resting showed a positive correlation in THYM and UCS.


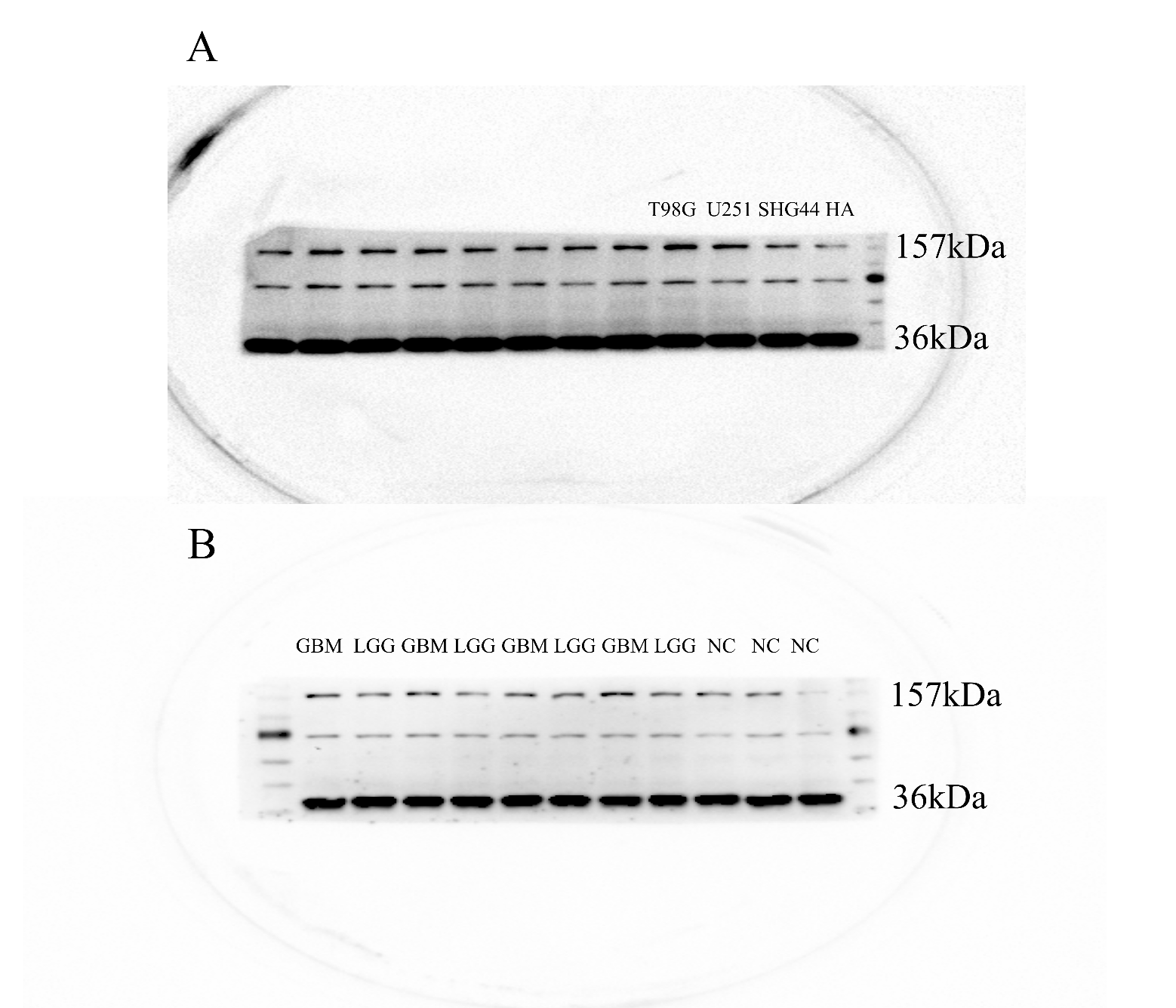


Supplementary Figure3 Expression of ATP7B in HA cell lines and glioma cell lines and in normal and glioma tissues.

A. Representative blot and summary data showed ATP7B protein levels in HA cell lines and glioma cell lines (n = 16).

B. Representative blotting and summary data showed ATP7B protein levels in normal and glioma tissues (n = 11).
